# Engineering CD276/B7-H3-targeted antibody-drug conjugates with enhanced cancer-eradicating capability

**DOI:** 10.1016/j.celrep.2023.113503

**Published:** 2023-11-28

**Authors:** Yang Feng, Jaewon Lee, Liping Yang, Mary Beth Hilton, Karen Morris, Steven Seaman, Veera V. Shivaji R. Edupuganti, Kuo-Sheng Hsu, Christopher Dower, Guojun Yu, Daeho So, Pradip Bajgain, Zhongyu Zhu, Dimiter S. Dimitrov, Nimit L. Patel, Christina M. Robinson, Simone Difilippantonio, Marzena Dyba, Amanda Corbel, Falguni Basuli, Rolf E. Swenson, Joseph D. Kalen, Sreedhar Reddy Suthe, Myer Hussain, James S. Italia, Colby A. Souders, Ling Gao, Martin J. Schnermann, Brad St. Croix

**Affiliations:** 1 Tumor Angiogenesis Unit, Mouse Cancer Genetics Program (MCGP), Center for Cancer Research (CCR), National Cancer Institute (NCI), National Institutes of Health (NIH), Frederick, MD 21702, USA; 2 Basic Research Program, Frederick National Laboratory for Cancer Research (FNLCR), Leidos Biomedical Research, Inc., Frederick, MD 21702, USA; 3 Organic Synthesis Section, Chemical Biology Laboratory, CCR, NCI, Frederick, MD 21702, USA; 4 Protein Interactions Section, Cancer and Inflammation Program, NCI, NIH, Frederick, MD 21702, USA; 5 Small Animal Imaging Program, FNLCR, Leidos Biomedical Research, Inc., Frederick, MD 21702, USA; 6 Animal Research Technical Support, FNLCR, Leidos Biomedical Research, Inc., Frederick, MD 21702, USA; 7 Biophysics Resource in the Center for Structural Biology, NCI, NIH, Frederick, MD, USA; 8 Invention Development Program, Technology Transfer Center, NCI, Frederick, MD 21701, USA; 9 Chemistry and Synthesis Center, National Heart, Lung, and Blood Institute, NIH, Rockville, MD 20850, USA; 10 BrickBio, Woburn, MA 01801, USA; 11 Veterans Affairs Long Beach Healthcare System, Long Beach, CA 90822, USA; 12 Present address: Center for Antibody Therapeutics, University of Pittsburgh School of Medicine, Pittsburgh, PA 15261, USA; 13 Lead contact

## Abstract

CD276/B7-H3 represents a promising target for cancer therapy based on widespread overexpression in both cancer cells and tumor-associated stroma. In previous preclinical studies, CD276 antibody-drug conjugates (ADCs) exploiting a talirine-type pyrrolobenzodiazepine (PBD) payload showed potent activity against various solid tumors but with a narrow therapeutic index and dosing regimen higher than that tolerated in clinical trials using other antibody-talirine conjugates. Here, we describe the development of a modified talirine PBD-based fully human CD276 ADC, called m276-SL-PBD, that is cross-species (human/mouse) reactive and can eradicate large 500–1,000-mm^3^ triple-negative breast cancer xenografts at doses 10- to 40-fold lower than the maximum tolerated dose. By combining CD276 targeting with judicious genetic and chemical ADC engineering, improved ADC purification, and payload sensitivity screening, these studies demonstrate that the therapeutic index of ADCs can be substantially increased, providing an advanced ADC development platform for potent and selective targeting of multiple solid tumor types.

## In brief

Off-target toxicities are a major obstacle to effective antibody-drug conjugate (ADC) therapy. By combining various genetic and biochemical approaches with payload sensitivity monitoring, Feng et al. demonstrate that the therapeutic window of talirine-based ADCs can be substantially improved, enabling the development of highly effective CD276-targeted ADCs with tumor-eradicating potential.

## Graphical Abstract

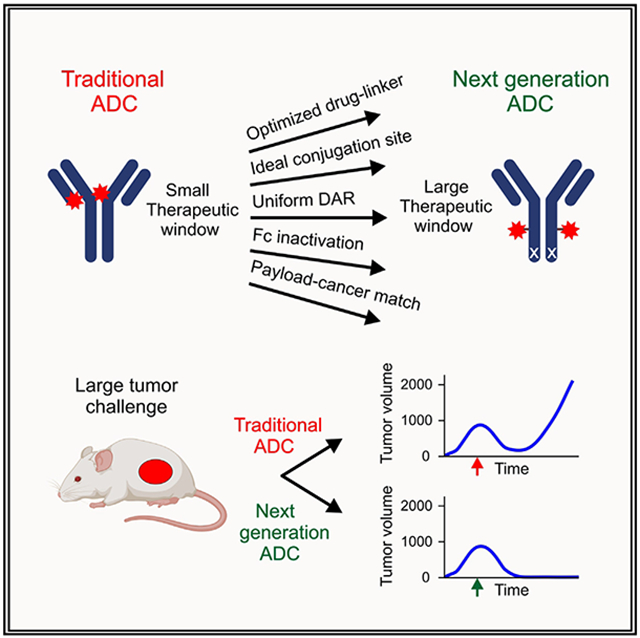

## INTRODUCTION

Monoclonal antibody (mAb) technology is revolutionizing cancer treatment by providing highly specific drugs with reduced side effects. Naked mAbs, like trastuzumab, which hinder receptor function or activate immune cells, are now essential tools in the oncologist’s toolkit. Despite their significance, mAbs often offer only modest short-term benefits to patients with widespread cancer. To enhance mAb effectiveness, there is a growing interest in equipping them with small-molecule drugs, giving rise to the prominence of antibody-drug conjugates (ADCs) as a crucial therapeutic class.^1^ For instance, trastuzumab emtansine (T-DM1; Kadcyla), an anti-HER2 ADC linked to DM1, surpasses the potency of its parent antibody and gained US Food and Drug Administration (FDA) approval in 2013 for treating HER2-positive breast cancer.^2^ Currently, the US FDA has approved 12 ADCs for clinical use.^3^ While these approvals underscore the significance of ADCs, challenges persist in achieving an optimal therapeutic index.^4^ Unfortunately, most clinically approved ADCs improve overall or progression-free survival of patients with cancer by only a few months compared with conventional standard-of-care chemotherapy.^5,6^ Furthermore, many ADCs target antigens overexpressed in a limited number of patients. For example, the target of trastuzumab, HER2, is amplified in the tumor cells of approximately 20% of patients with metastatic breast cancer.^7^ Finally, a meta-analysis of toxicities associated with ADCs in clinical trials revealed a stark reality: while ADC efficacy relies on target-antigen expression, most dose-limiting toxicities are target-antigen independent.^8-11^ Indeed, dose-limiting toxicities typically reflect those of the ADC drug payload.^8,12^ Therefore, to advance this field and reduce cancer mortality, efforts to identify optimal cancer targets and enhance the ADC therapeutic window are urgently needed. To improve ADC tolerability, a better understanding of the underlying factors that cause ADC off-target toxicities is needed, along with effective reengineering approaches to mitigate them.

**Figure 1.**
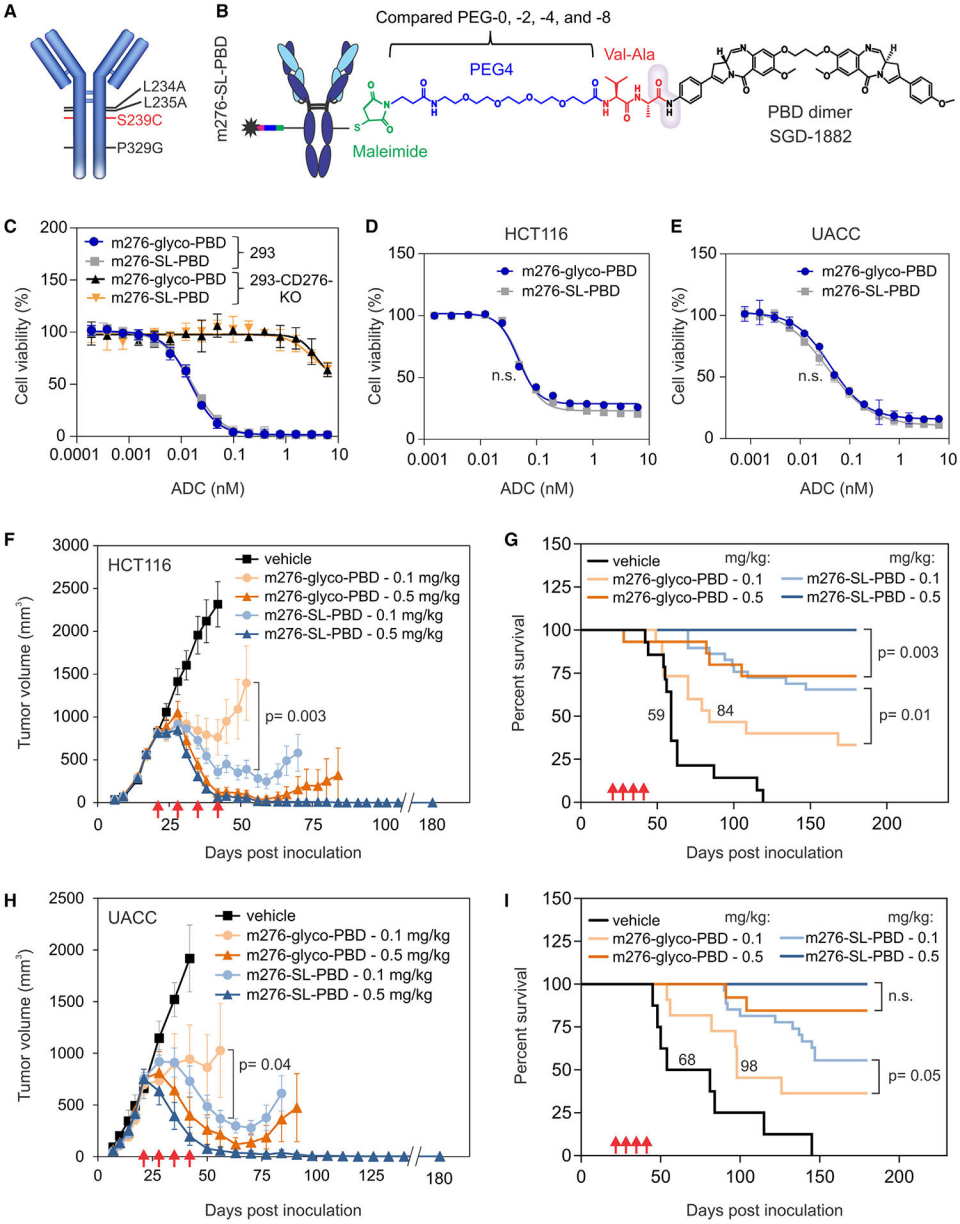
m276-SL-PBD structure and activity comparison with m276-glyco-PBD (A) Amino acid substitutions in m276-SL. (B) Chemical structure of m276-SL-PBD linker and warhead: maleimide (green), PEG-4 spacer (blue), and cathepsin-B-cleavablevaline-alanine dipeptide (red). The gray cloud indicates the cleavable amide group. (C–E) Cell viability assays were used to measure the activity of m276-SL-PBD and m276-glyco-PBD against the parent 293 (CD276 wild type) or 293-CD276 KO (CD276 knockout) (C), HCT116 colon cancer (D), or UACC melanoma cells (E). Error bars denote SD. (F–I) Subcutaneous growth of HCT-116 (F) and UACC (H) tumors and corresponding Kaplan-Meier survival curves (HCT116, G; UACC, I). ADC treatments were initiated when tumors reached an average size of ~750 mm^3^ and were administered on the days shown (red arrows); n = 8–30/group; p values: t test (F and H) and log-rank test (G and I). Median survival is indicated for arms with <50% of animals alive at study end. Error bars denote SEM. n.s., non-significant.

Our earlier work reported the creation of ADCs targeting CD276 (B7-H3), a cell-surface molecule widely overexpressed in cancer cells and tumor-associated stromal cells across various cancers, including breast, colon, and lung carcinomas.^13,14^ Overexpression of CD276 in tumors is widely associated with a worse prognosis. Although the functions of CD276 remain largely unclear, accumulating evidence suggests its involvement in promoting immunosuppression, partly through T cell exclusion.^15-17^ In tumor-associated stromal cells, CD276 is highly overexpressed on co-opted endothelium and in the neovasculature of pathological but not physiological angiogenesis, helping alleviate concerns of collateral damage from CD276-targeted therapy.^13,14^ Our previously described CD276 ADC, herein called m276-glyco-PBD, employed a fully human CD276 mAb, called m276.^14^ This ADC was armed via a modified carbohydrate side chain to a pyrrolobenzodiazepine (PBD) dimer (SGD-1882), the same payload used in the drug-linker talirine (Figure 1B). Similar to the parent m276 mAb, this ADC demonstrates high-affinity binding to both human and mouse CD276 (K_D_ 29 and 24 nM for hCD276 and mCD276, respectively) and, in preclinical tumor studies, displayed minimal toxicities at doses that proved highly efficacious. PBD dimers, known for their potency, are DNA cross-linking agents that are insensitive to P-glycoprotein (P-gp/ABCB1/MDR1) drug efflux, can target both dividing and non-dividing cells, have potential for evading DNA repair mechanisms, and, in contrast to more common payloads like Monomethyl auristatin E (MMAE), are effective against both tumor cells and tumor-associated stroma.^14^ Free PBDs, including SJG-136, which has been tested in clinical trials, exhibit a degree of selectivity toward certain tumor types.^18^ While further studies are required to understand the mechanistic basis of cancer cells’ heightened sensitivity to PBDs compared with normal cells, this sensitivity serves as an additional safeguard against toxicities caused by unintentional ADC drug shedding in the circulation. In April 2021 the CD19-targeted loncastuximab tesirine became the first PBD-armed ADC to be clinically approved in the United States and is used to treat relapsed or refractory diffuse large B cell lymphoma.^19^

Typically, preclinical ADC therapeutic studies in mice focus on treating small subcutaneous tumors ranging from 100 to 200 mm^3^ in size.^14,20,21^ However, tumors or metastases found in patients with cancer are frequently closer to a volume of 1,000 mm^3^ by the time they are detectable.^22,23^ Success in treating larger tumors is crucial, because acquired drug resistance in tumors depends on both the mutation rate and the absolute tumor cell number.^24,25^ Furthermore, in preclinical studies, m276-glyco-PBD required a dose of 500–1,000 μg/kg to achieve maximal response, which is near the maximum tolerated dose (MTD) in mice, while the MTD in human clinical trials of other SGD-1882 PBD-linked ADCs, such as SGN-CD33A and SGN-CD70A, was in the 25–50 μg/kg range.^26-28^ Given that off-target toxicities are far more prominent than on-target toxicities across the ADC field, including those caused by PBD-based ADCs,^29^ we posit that significant enhancements in ADC design and production are imperative for these therapeutic agents to achieve their maximum potential. In the current study, we adopt a multifaceted approach, involving genetic and chemical reengineering of both the antibody and the drug linker, to create a fully optimized CD276 ADC that substantially mitigates off-target toxicities. The immunoconjugate, called m276-SL-PBD, is a CD276 mouse-human cross-species-reactive ADC with a substantially improved therapeutic window and widespread effectiveness against various solid tumor types.

## RESULTS

### Enhancing ADC tolerability through Fc-domain engineering

Our previously described PBD-linked CD276 ADC, m276-glyco-PBD, utilized a modified carbohydrate at N297 of the m276 anti-CD276 antibody heavy chain for precise site-specific drug conjugation.^14^ While glycan conjugation provides a facile method to create ADCs, it also raises concerns about exposing the PBD on the surface, potentially leading to non-specific binding as observed for other hydrophobic ADCs that bind non-specifically to cells of the reticuloendothelial system.^30^ In addition, hydrophobic ADCs are prone to aggregation, promoting non-specific binding and phagocytic cell uptake.^30-34^ PBD glycoconjugation also requires glycosidase pretreatment to remove most of the carbohydrate side chain, potentially compromising ADC stability^35^ and complicating manufacturing scale-up. To address these concerns, we engineered a free cysteine into a protected site on the antibody heavy chain (S239C) for site-specific maleimide-mediated drug conjugation (Figure 1A). Conjugation of the PBD drug linker at S239C of the heavy chain has been shown to increase ADC solubility, drastically reducing aggregation, likely because the hydrophobic drug is partly buried in a pocket of the Fc domain.^36,37^ Shielding from the nearby hydrophilic carbohydrate side chain at N297 may also help reduce surface hydrophobicity. Conjugation to S239C also protects the payload from premature release through a retro-Michael reaction, preventing the exposed maleimide-drug linker from encountering scavenging sulfhydryls in the serum, such as cysteine-34 in albumin.^36,38^ Finally, S239C linkage helps prevent cleavage of the valine-alanine (VA) drug linker by circulating enzymes,^39^ averting the potential problem of premature drug shedding.

Another concern for toxicity is that Fc domains may enable ADCs to bind Fc-receptor-positive cells of the innate immune system and kill them.^40^ To mitigate this reactivity, we engineered three mutations, L234A, L235A, and P329G (collectively referred to as LALAPG), into the m276 CD276 antibody heavy chains, which block Fcγ receptor (FcγR) binding without altering antibody stability or immunogenicity (Figure 1A).^41-43^ The reengineered ADC, called m276-SL-PBD (for S239C, LALAPG), showed diminished FcγR binding, maintained indistinguishable CD276 binding compared with the parent m276 antibody, and displayed typical pharmacokinetic (PK) properties *in vivo* (Figure S1).

### Optimization of the drug linker

The PBD dimer payload utilized in our CD276 ADC, known as SGD-1882 (Figure 1B), is identical to that used in talirine, a drug linker tested on several ADCs in clinical trials.^44^ However, talirine, which lacks a hydrophilic polyethylene glycol (PEG) group, is highly hydrophobic and challenging to dissolve, necessitating conjugation in 50% propylene glycol. In an effort to enhance ADC performance, we compared SGD-1882 drug linkers without PEG (PEG-0) with those incorporating various numbers of hydrophilic PEG units (PEG-2, -4, and -8) inserted between the maleimide and the cleavable VA dipeptide (Figures 1B and S2A). All drug linkers with PEG displayed increased solubility compared with the PEG-0 drug linker (Figure S2B), facilitating an improved conjugation process and enabling the production of ADCs with higher purity and yields. Because the insertion of a PEG group could potentially push the hydrophobic PBD further from the ADC surface, potentially leading to increased non-specific uptake and toxicity *in vivo*, we also assessed the impact of PEG length on overall ADC tolerability *in vivo*. Body weight measurement following high-dose ADC administration revealed that the PEG-0 and PEG-4-containing ADCs were both well tolerated, while the PEG-2 and PEG-8 ADCs were consistently more toxic (Figure S2C). While the reason for the improved enhanced performance of the intermediate-length PEG-4 requires further structural analysis, one possibility is that it provides an optimal balance between overall drug-linker solubility and surface hydrophobicity induced by the protruding PBD. Nonetheless, as PEG-4 improved the drug linker’s solubility without adversely affecting ADC toxicity *in vivo*, we opted for PEG-4 in m276-SL-PBD for all subsequent evaluations.

Next, using an HCT116 colon tumor xenograft model, we compared the anti-tumor efficacy of m276-SL-PBD with that of DS-7300a, a CD276 ADC that recently entered clinical trials and employs a DNA topoisomerase I inhibitor payload.^20^ We also labeled the m276 antibody with the PBD drug linker tesirine, following the same method used for the clinically approved CD19-targeted loncastuximab tesirine ADC. As shown in Figure S3, while 10 mg/kg DS-7300a and 0.5 mg/kg m276-tesirine only induced tumor growth delays, 0.5 mg/kg m276-SL-PBD resulted in a complete and sustained tumor response, leading us to conclude that m276-SL-PBD is a much more potent ADC. Based on these findings, we selected m276-SL-PBD containing the SGD-1882 PBD from talirine as our lead ADC.

### Comparison of m276-SL-PBD with m276-glyco-PBD

Next, we conducted *in vitro* cytotoxicity assays to compare m276-SL-PBD with m276-glyco-PBD. The “SL” modifications in our ADC were designed to minimize non-specific binding to phagocytic cells *in vivo* with no anticipated changes in potency against target cells *in vitro*. As expected, m276-SL-PBD selectively killed CD276^+^ HEK293 (293), HCT116 colon cancer, and UACC melanoma cells (Figure S1A) with an IC_50_ in the low picomolar range, indistinguishable from our previous m276-glyco-PBD ADC (Figures 1C-1E). Disruption of CD276 in 293 cells using CRISPR-Cas9 rendered the cells over 100-fold more resistant to both CD276 ADCs, highlighting the target dependency of these ADCs. Although non-specific cell killing at high drug doses (>1 nM) was observed, this was likely due to low-level uptake through pinocytosis (Figure 1C). While m276-SL-PBD and m276-glyco-PBD exhibited similar *in vitro* cytotoxicity, *in vivo* testing demonstrated enhanced potency of m276-SL-PBD against large HCT116 and UACC (~1,000 mm^3^) primary tumors following treatment with 500 or 100 μg/kg once per week for 4 weeks (Figures 1F-1I). In these studies, all mice treated with 500 μg/kg m276-SL-PBD displayed complete regression and remained tumor free 6 months post treatment (Figures 1G and 1I). We conclude that m276-SL-PBD is more potent *in vivo* than m276-glyco-PBD, despite both ADCs having a drug-to-antibody ratio (DAR) of about 1.9 and indistinguishable *in vitro* cytotoxic activities. The increased efficacy of m276-SL-PBD may enable similar activity at lower ADC doses, thereby further reducing toxicity through dose reduction.

### Enhanced tumor targeting with S239C site-specific drug labeling

**Figure 2.**
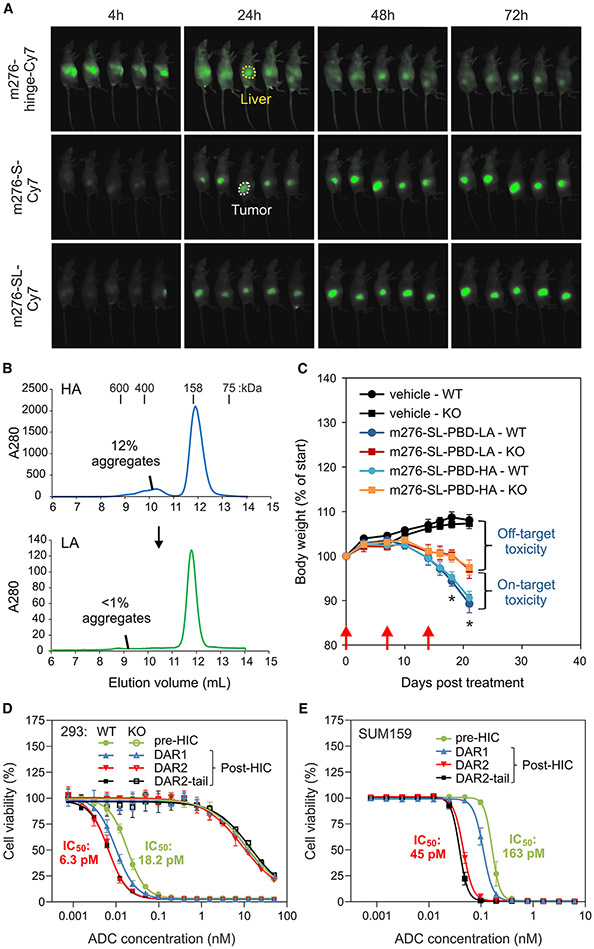
Factors influencing m276-SL-PBD activity and toxicity (A) *In vivo* fluorescence imaging of Cy7-labeled m276 antibodies in JIMT tumor-bearing mice at 4, 24, 48, and 72 h post injection. Side-view images of the tumor flank are shown. An example of tumor and liver fluorescence is highlighted (white and yellow regions of interest [ROIs], respectively). (B) SEC monitoring of m276-SL-PBD samples with high-aggregate (HA) and low-aggregate (LA) composition pre- and post purification by SEC. Size standards are shown at the top. (C) Body weights in CD276-WT and -KO mice after three treatments (red arrows) with 2 mg/kg of LA and HA ADC samples from (B). Student’s t test; *p < 0.05 for m276-SL-PBD-LA in WT versus KO and m276-SL-PBD-HA in WT versus KO; n = 8–15/group. (D and E) Cell viability assays measured the activity of m276-SL-PBD pre- and post-HIC purification against HEK293 CD276-WT, CD276-KO (D), or CD276^+^ SUM159 breast cancer cells (E). HIC-enriched DAR1, DAR2, and DAR2-tail fractions were tested (see Figure S5B). Error bars denote SD.

The m276-SL-PBD described here utilized site-specific PBD conjugation at an engineered free cysteine (S239C). However, all 12 clinically approved ADCs have employed random conjugation at either lysines or cysteines, the latter of which were used to arm the CD19-targeted PBD conjugate loncastuximab tesirine.^45,46^ To assess whether site-specific labeling at S239C could enhance the therapeutic index, we employed a hydrophobic farred fluorophore, Lumiprobe Cy7 maleimide, as a PBD surrogate to label m276 either stochastically on endogenous cysteines or site specifically at S239C for subsequent *in vivo* immunofluorescence tracing. For random Cy7 conjugation, we labeled the parent m276 antibody, while for site-specific conjugation at S239C, we labeled both m276-S (containing S239C only) and m276-SL (containing S239C and LALAPG) to assess the impact of Fc inactivation on biodistribution *in vivo*. Adjusting the labeling conditions to ensure similar fluorophore amounts on all three CD276 antibodies, we injected 50 μg (~2 mg/kg) of each antibody-fluorophore conjugate (AFC) into tumor-bearing mice and performed fluorescence imaging *in vivo* and *ex vivo* after 72 h. These studies revealed a striking increase in tumor-to-liver ratio in both AFCs with site-specific drug labeling at S239C (Figures 2A and S4). Based on this, we conclude that S239C site-specific labeling plays a dominant role in decreasing non-specific uptake in normal tissues.

### Elevated ADC potency through enhanced purification

Next, we assessed if further improvements in the therapeutic window could be achieved through optimization of the purification strategy. Initially, we examined the impact of ADC soluble aggregates on toxicity *in vivo* to determine if a standard cutoff of <5% aggregates was suitable for m276-SL-PBD, as aggregates can potentially lead to increased toxicity *in vivo*.^47^ We selected a batch of m276-SL-PBD with an unusually high level of soluble aggregates (13%) and used size-exclusion chromatography (SEC) to eliminate the aggregates from a portion of the material, yielding a sample with low aggregates (<1%) for comparative analysis. Subsequently, to assess toxicity, we administered the samples with high and low aggregates to non-tumor-bearing mice at a dose sufficient to induce body weight loss (i.e., 2 mg/kg once per week for 3 weeks). While both high- and low-aggregate ADCs decreased body weight, surprisingly, differences between the groups were indistinguishable (Figures 2B and 2C). By conducting these ADC studies in CD276-wild-type (WT) and -knockout (KO) mice, we were also able to compare on-target versus off-target toxicity. High-dose m276-SL-PBD treatment led to ~17% body weight loss in CD276-WT mice and ~10% in CD276-KO mice. This suggests that, while some ADC toxicity is on-target, a significant portion remains off-target.

ADC payloads are typically hydrophobic, as this property enables the drug, upon lysosomal release from the antibody, to diffuse across intracellular membranes and reach its target: double-stranded DNA in the case of PBD. Diffusion across cellular membranes is also critical for antigen-independent bystander killing, addressing the problem of tumor target heterogeneity.^48^ However, the increased surface hydrophobicity due to hydrophobic payloads may contribute to toxicity by promoting non-specific uptake *in vivo*. To assess this, hydrophobic-interaction chromatography (HIC) was used to evaluate m276-SL-PBD before and after drug conjugation. Surprisingly, analytical HIC revealed that approximately 25% of the purified m276-SL-PBD eluted at the same time as the parent antibody, suggesting the presence of unmodified parent antibody (DAR = 0), which was subsequently confirmed by mass spectrometry (MS) analysis (Figure S5). Because further attempts to reduce the unmodified antibody (DAR = 0) fraction through reaction condition optimization were unsuccessful, large-scale preparative HIC purification was performed to eliminate the parent (DAR = 0) fraction. Following HIC purification, fractions surrounding the DAR1 peak, the DAR2 peak, and the DAR2 tail (representing low, intermediate, and highly hydrophobic samples, respectively) were isolated (Figure S5B). MS, reverse-phase high-performance liquid chromatography (RP-HPLC), and analytical HIC analyses confirmed the effective labeling of the DAR2 samples and the removal of unlabeled antibody (Figures S5B and S5C). MS analysis did not detect any free drug in any of the fractions. In the DAR2 tail, an ADC was identified with increased levels of hydrolyzed (functional) PBD attached to the antibody, which may have contributed to the increased retention time of this fraction.

Cell viability assays were conducted to assess the efficacy of unfractionated and purified samples. Remarkably, when tested against CD276^+^ 293 and SUM159 cells, these assays demonstrated 2.9- to 3.6-fold higher killing activity in the HIC DAR2-enriched fractions compared with the unfractionated sample. The DAR1-enriched sample, as anticipated, was less potent than the DAR2 samples but still exhibited higher potency than the unfractionated sample (Figures 2D and 2E). Lower activity of the unfractionated material may be partially attributed to competition from unmodified parent antibody. However, the observed killing in the non-purified sample was lower than expected based on its DAR0 contamination (~25%), suggesting that the added HIC purification step may have unanticipated benefits. Interestingly, despite differences in apparent hydrophobicity as indicated by HIC, higher doses of DAR1, DAR2, and DAR2 tail demonstrated similar levels of non-specific killing when tested against target-negative CD276-KO cells (Figure 2D). This suggests that the enhanced post-purification potency is not due to increased non-specific binding and uptake by pinocytosis. While HIC is mostly used as an analytical tool to assess DARs in the ADC field, our unexpected finding that significant improvement in ADC potency can be achieved through HIC purification of the DAR2 fraction led us to incorporate preparative HIC as an essential step in our large-scale ADC production protocol.

### Assessing ADC biodistribution using zirconium-89 PET imaging

**Figure 3.**
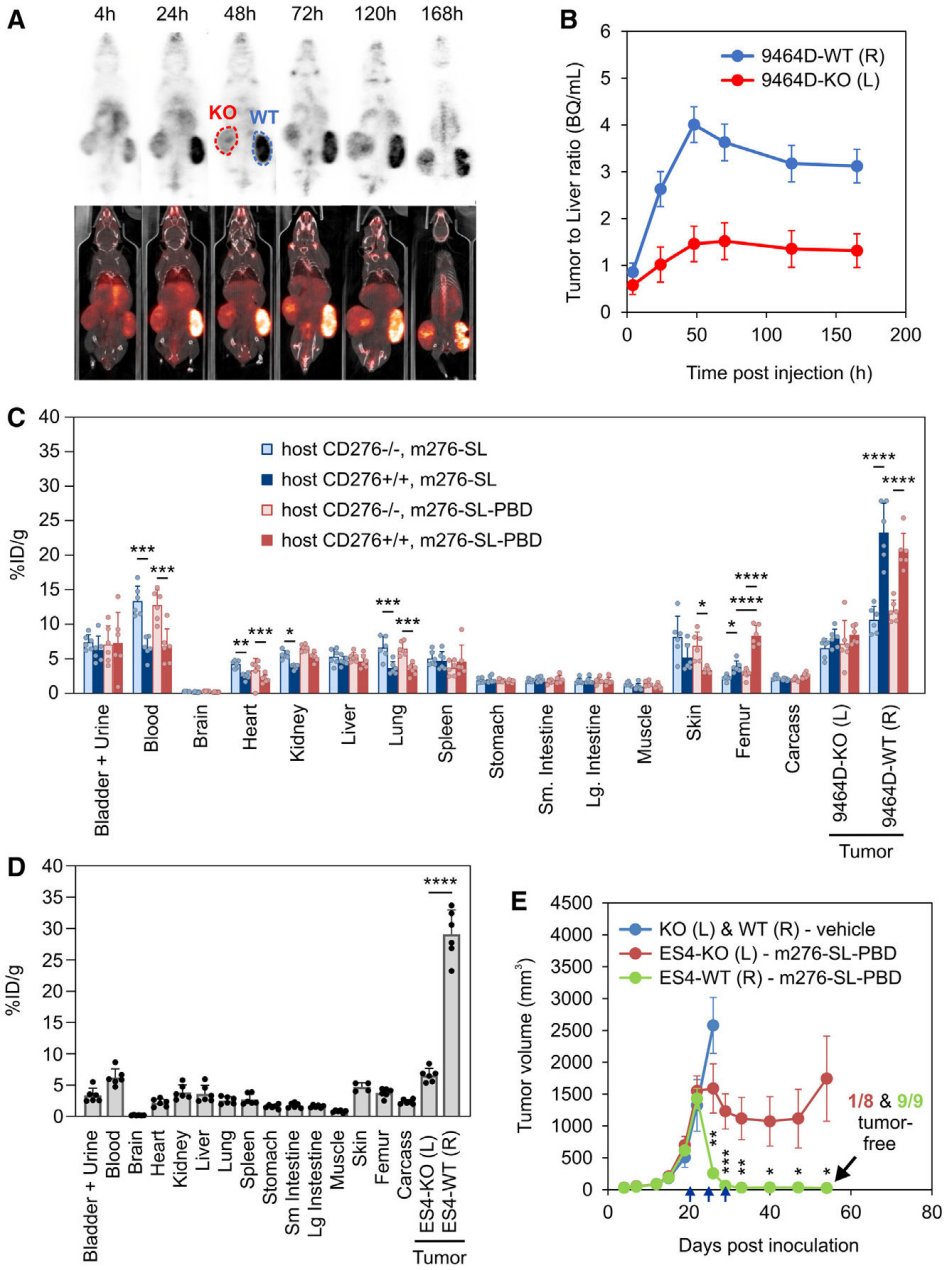
*In vivo* biodistribution of [^89^Zr]Zr-DFO-m276-SL and [^89^Zr]Zr-DFO-m276-SL-PBD (A) PET imaging at ~4, 24, 48, 72, 120, and 168 h post injection of 0.5 mg/kg [^89^Zr]Zr-DFO-m276-SL-PBD ADC into mice with 9464D-CD276-WT (right flank) and 9464D-CD276-KO (left flank) tumors. (B) Quantification of tumor/liver labeling ratios from the PET study in (A). Error bars denote SD. (C) Biodistribution analysis 48 h post intravenous injection of [^89^Zr]Zr-DFO-m276-SL antibody or [^89^Zr] Zr-DFO-m276-SL-PBD ADC in CD276-wild-type (+/+) or -knockout (−/−) C57BL/6 mice. 9464D CD276-WT (right flank) and CD276-KO (left flank) tumors from the same mice were included for comparison. A one-way ANOVA determined p values between groups. Error bars denote SD. (D) Biodistribution analysis 48 h post injection of [^89^Zr]Zr-DFO-m276-SL-PBD ADC in athymic nude mice with ES4-CD276-WT (right flank, R) and ES4-CD276-KO (left flank, L) Ewing’s tumors. T test determined p values between WT and KO ES4 tumors. Error bars denote SD. (E) Subcutaneous growth of ES4-CD276-WT (right flank, R) or ES4-CD276-KO (left flank, L) Ewing’s tumors after m276-SL-PBD treatment. Treatments (blue arrows) were initiated when tumors reached an average size of ~750 mm^3^; n = 6–9/group. A t test determined p values between WT and KO ES4 tumors at the indicated time points. Error bars denote SEM. p values for (C)–(E): *p ≤ 0.05, **p ≤ 0.01, ***p ≤ 0.001, and ****p ≤ 0.0001.

Having established an optimized protocol for ADC purification, next we evaluated the biodistribution of the m276-SL parent antibody and m276-SL-PBD ADC in tumor-bearing mice using zirconium-89 (^89^Zr) positron emission tomography (PET) imaging. To evaluate specificity, the CD276 gene was disrupted in the 9464D murine neuroblastoma cell line using CRISPR-Cas9 (Figure S1A). Subsequently, 9464D-WT (CD276 WT) and 9464D-KO (CD276 KO) tumor cells were injected subcutaneously on opposite flanks of syngeneic C57BL/6 mice. When tumors reached an average size of 1,000 mm^3^, the mice received intravenous injections of 0.5 mg/kg of either [^89^Zr]Zr-DFO-m276-SL antibody or [^89^Zr]Zr-DFO-m276-SL-PBD ADC, and PET imaging was performed at approximately 4, 24, 48, 72, 120, and 168 h post inoculation. These studies revealed peak tumor/liver intensity ratios in WT tumors around 48 h post injection (Figures 3A and 3B). Next, we analyzed the radioactivity levels (percentage injected dose per gram of tissue) in various tissues from CD276-WT or -KO mice, including 9464D-WT and -KO tumors, 48 h post injection of the zirconium-89-labeled antibody or ADC. As anticipated, the lowest levels of ADC were observed in the brain due to the blood-brain barrier. Levels of both [^89^Zr]Zr-DFO-m276-SL and [^89^Zr]Zr-DFO-m276-SL-PBD increased approximately 2-fold in the blood of CD276-KO versus -WT mice, suggesting that a decrease in on-target binding may contribute to the elevated blood levels (Figure 3C). Except for the femur, where shed zirconium-89 is known to accumulate,^49^ no significant increase in binding to tissues with the [^89^Zr] Zr-DFO-m276-SL-PBD ADC compared with the parent [^89^Zr]Zr-DFO-m276-SL antibody was observed. This indicates that any potential increase in non-specific uptake *in vivo* caused by the hydrophobic PBD was not detectable by this method.

In a second model, CD276-WT athymic nude mice were subcutaneously challenged with ES4 Ewing’s sarcoma by implanting CD276-WT and -KO tumor cells on opposite flanks. When tumor volume reached an average of 300 mm^3^, the mice received intravenous injections of 0.5 mg/kg [^89^Zr]Zr-DFO-m276-SL-PBD, and tissues were removed 48 h later for biodistribution analysis. ES4-WT tumors accumulated the most drug (29% injected dose per gram of tissue [ID/g]), followed by the ES4-KO tumors (6.8% ID/g) and then blood (6.2% ID/g) (Figure 3D). In a parallel therapeutic study, mice were treated with the m276-SL-PBD ADC when tumors reached 1,000 mm^3^ in size. In this study, only ES4-WT tumors on the right flank could be completely eradicated by the ADC, while the ES4-KO tumors on the left flank exhibited a partial response, presumably due to ADC targeting of CD276^+^ tumor-associated stromal cells (Figure 3E). These findings collectively suggest that CD276 expression in tumor-associated stroma alone is insufficient for a complete response and that tumor eradication in this model requires target co-expression in cancer cells.

### Unveiling ADC hypersensitivity: Insights from cancer sensitivity screening

**Figure 4.**
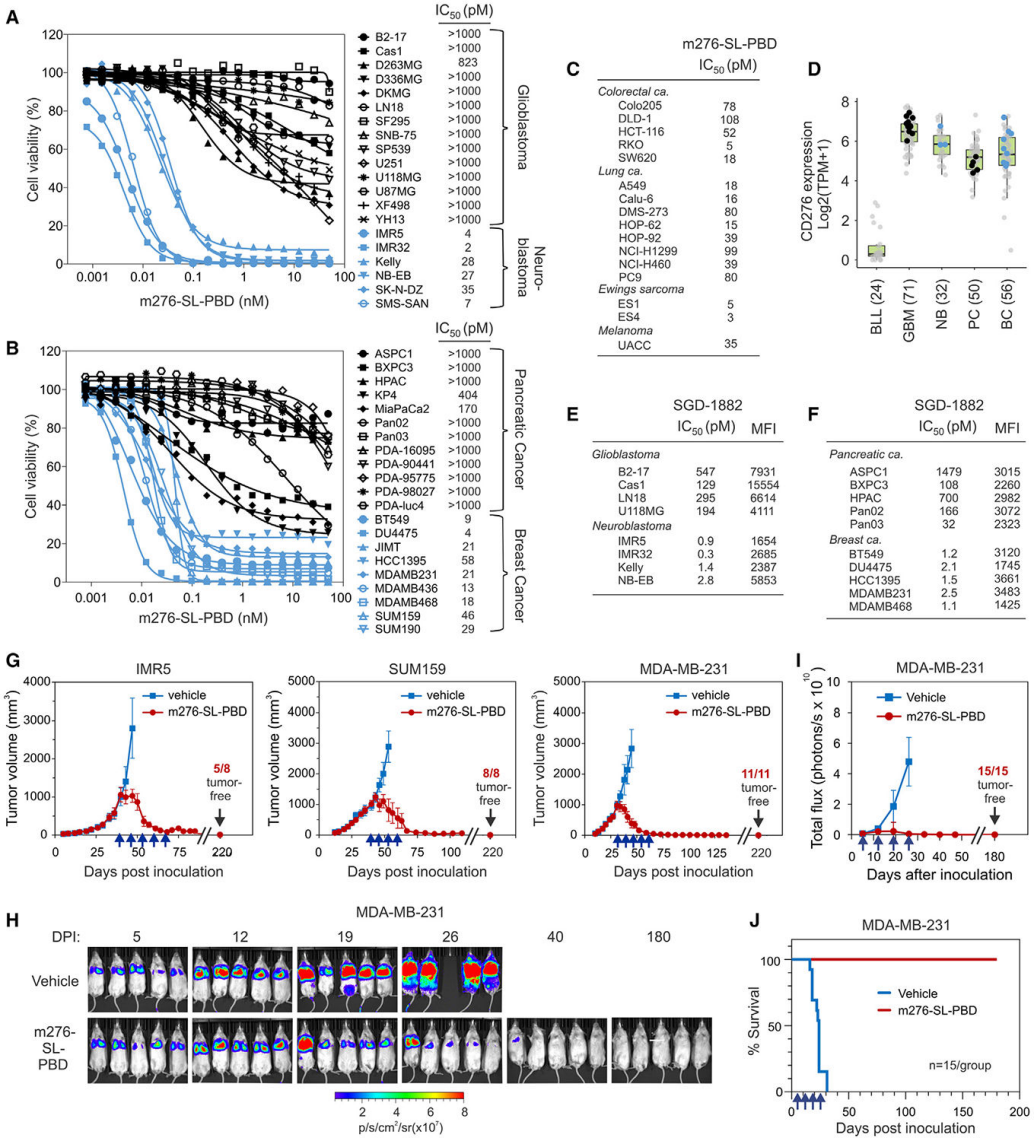
Sensitivity to m276-SL-PBD across cancer types (A and B) Cell viability assays measured m276-SL-PBD activity against glioblastoma/neuroblastoma (A) and pancreatic/breast cancer (B) cell lines. IC_50_ values are in the key. Error bars were omitted for clarity; SD was always <10%. (C) Table of IC_50_ values from cell viability assays showing the relative sensitivity of cancer cell lines to m276-SL-PBD. (D) CD276 mRNA expression in CD276-low B cell lymphocytic leukemia (BLL) and CD276-high glioblastoma (GBM), neuroblastoma (NB), pancreatic cancer (PC), and breast cancer (BC) cell lines using the database: DepMap mRNA expression. The number of cancer cell lines in each group is indicated in parentheses. Sensitive (blue dots) and resistant (back dot) cell lines used in the cell viability assays (A and B) are highlighted. (E and F) Tables showing glioblastoma/neuroblastoma (E) or pancreatic/breast cancer (F) cell line sensitivity (IC_50_ values from cell viability assays) after SGD-1882-free drug treatment and CD276 surface expression levels (mean fluorescence intensity; MFI) as measured by flow cytometry. (G) Subcutaneous (IMR5) and orthotopic (SUM159 and MDA-MB-231) tumor volumes following ADC treatment. Treatments with 0.5 mg/kg m276-SL-PBD (blue arrows) were initiated when tumors reached ~1,000 mm^3^; n = 8–10/group. Error bars denote SEM. (H) Bioluminescence imaging monitored systemic MDA-MB-231-luc tumor burden following intravenous injection into NRG mice, followed by randomization and treatment with vehicle or m276-SL-PBD. Representative mice shown (n = 15/group; DPI, days post inoculation). (I) Quantification of tumor burden from the MDA-MB-231 metastasis study in (H). Error bars denote SD. (J) Kaplan-Meier survival curves for the MDA-MB-231-luc metastases study in (H).

By comparing CD276-positive cancer cell lines with CRISPR-Cas9-engineered isogenic CD276-KO controls, we verified that target expression is essential for potent (subnanomolar) activity of the m276-SL-PBD ADC, but not for the PBD-free drug SGD1882 (Figure S6). While testing m276-SL-PBD against 57 CD276-positive cell lines, all exhibiting similarly high levels of CD276, it became evident that the sensitivity of each cell line was also highly dependent on the cancer cell type. For instance, all 18 CD276^+^ glioblastomas were highly resistant to the ADC, with IC_50_ values greater than 1 nM, while all 6 CD276^+^ neuroblastomas were highly sensitive, with IC_50_ values generally much less than 1 nM (Figure 4A). Similarly, CD276-positive cell lines derived from pancreatic tumors tended to be relatively resistant, whereas those from breast cancers, colon cancers, lung cancers, and pediatric Ewing’s sarcoma were hypersensitive (Figures 4B and 4C). The resistance observed was not attributable to a lack of CD276 expression, as all tested cancer cell types displayed similar CD276 mRNA levels in the Cancer Dependency Map (DepMap) dataset and comparable cell surface protein levels as assessed by flow cytometry (Figures 4D-4F). Furthermore, neuroblastoma and breast cancer cell lines exhibited high sensitivity to free PBD (SGD-1882), whereas glioblastoma and pancreatic cell lines were more resistant (Figures 4E and 4F). The mechanisms underlying these large differences in sensitivity could be related to cell-type-specific variations in driver mutations, mitotic index, apoptotic sensitivity, DNA repair capacity, drug trafficking, drug metabolism, or efflux.^50,51^ Although further investigation is needed to fully comprehend the basis of this difference from PBDs, nevertheless, these findings suggest that *in vitro* sensitivity screening could serve as a valuable tool for identifying cancer types that are intrinsically hypersensitive, aiding personalized therapy and helping ensure an optimal therapeutic window for PBD-containing ADCs.

### Preclinical assessment demonstrates potent efficacy of m276-SL-PBD against xenograft tumors

Encouraged by the particularly high activity of m276-SL-PBD against neuroblastoma and breast cancer cells *in vitro*, our next step was to compare ADC activity *in vivo* against large 1,000-mm^3^ human tumor xenografts of each of these cancer types. As shown in Figure 4G, *in vivo* preclinical testing revealed potent activity of m276-SL-PBD ADCs against large (~1,000 mm^3^) subcutaneous IMR5 neuroblastoma and orthotopically implanted SUM159 or MDA-MB-231 breast tumors in the mammary fat pad. Notably, 100% of animals challenged with ER^−^, PR^−^ HER2^−^ SUM159 and MDA-MB-231 tumors exhibited complete and sustained tumor regression 6 months post treatment, suggesting that CD276 could be a critical target for triple-negative breast cancer (TNBC). Considering that metastases are the primary cause of breast cancer mortality, m276-SL-PBD was also tested against preestablished metastases derived from the luciferase-tagged MDA-MB-231-luc cell line. Five days following intravenous injection of tumor cells, ADCs were administered once per week for 4 weeks. Strikingly, the ADC was highly effective at eliminating metastases, with 100% of mice showing no signs of tumor 6 months post treatment (Figures 4H-4J).

**Figure 5.**
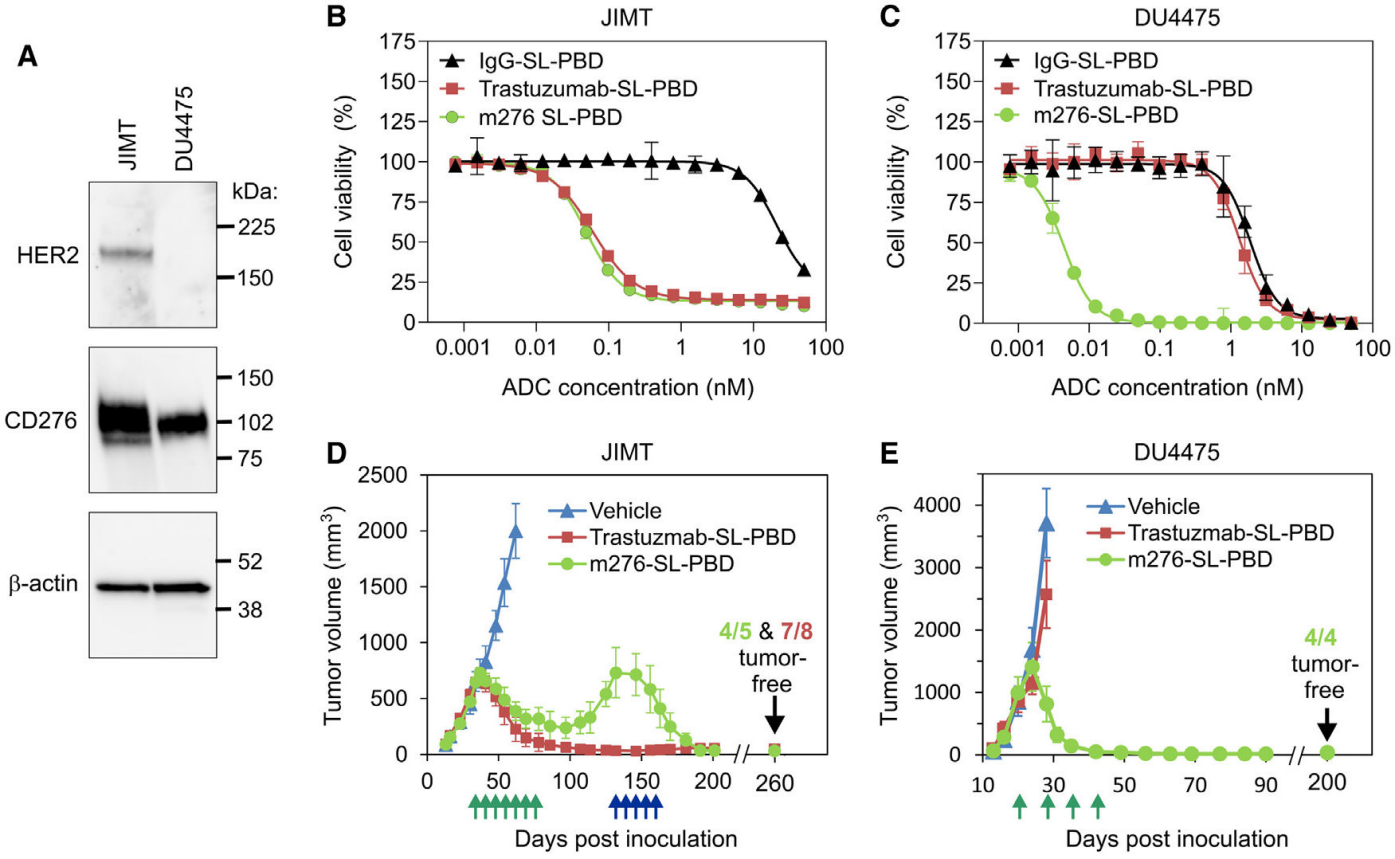
m276-SL-PBD targets CD276^+^ breast cancer independent of HER2 status (A) Western blot analysis of CD276 and HER2 levels in JIMT and DU4475 breast cancer cells. (B and C) Cell viability assays measured trastuzumab-SL-PBD and m276-SL-PBD activity against JIMT (B) and DU4475 (C) breast cancer cells. Non-specific IgG-SL-PBD served as a non-binding control. Error bars denote SD. (D and E) Orthotopic growth of JIMT (D) and DU4475 (E) breast tumors in response to trastuzumab-SL-PBD and m276-SL-PBD treatment. ADC treatments (0.5 mg/kg) were initiated when tumor volumes reached ~750–1,000 mm^3^ and administered on the days shown (green arrows). Relapsed tumors in the m276-SL-PBD group were re-treated five times with ADC (blue arrows) when tumor volumes reached an average of ~750 mm^3^; n = 5–8/group. Error bars denote SEM.

To assess the applicability of our conjugation approach to other ADCs, we generated trastuzumab-SL, an anti-HER2 antibody with the SL mutations, and conjugated it with our SGD-1882 PBD drug linker (Figures 1A and 1B). We then compared its performance with that of m276-SL-PBD. Beginning with *in vitro* testing against the HER2^+^/CD276^+^ JIMT-1 (JIMT) and the HER2^−^/CD276^+^ DU4475 breast cancer cell lines (Figure 5A), we found that both ADCs exhibited potent activity against the double-positive cells (Figure 5B). However, only m276-SL-PBD could effectively eliminate CD276^+^/HER2^−^ DU4475 (Figure 5C). Similarly, when tested *in vivo* against orthotopic breast tumors, again ADC activity was target dependent, with the trastuzumab-SL-PBD eliciting tumor regression only in the JIMT model, while the m276-SL-PBD was effective against both JIMT and DU4475 tumors (Figures 5D and 5E). Although some m276-SL-PBD-treated tumors relapsed in the JIMT study, they remained sensitive to re-treatment, resulting in an 80% long-term remission (Figure 5D).

**Figure 6.**
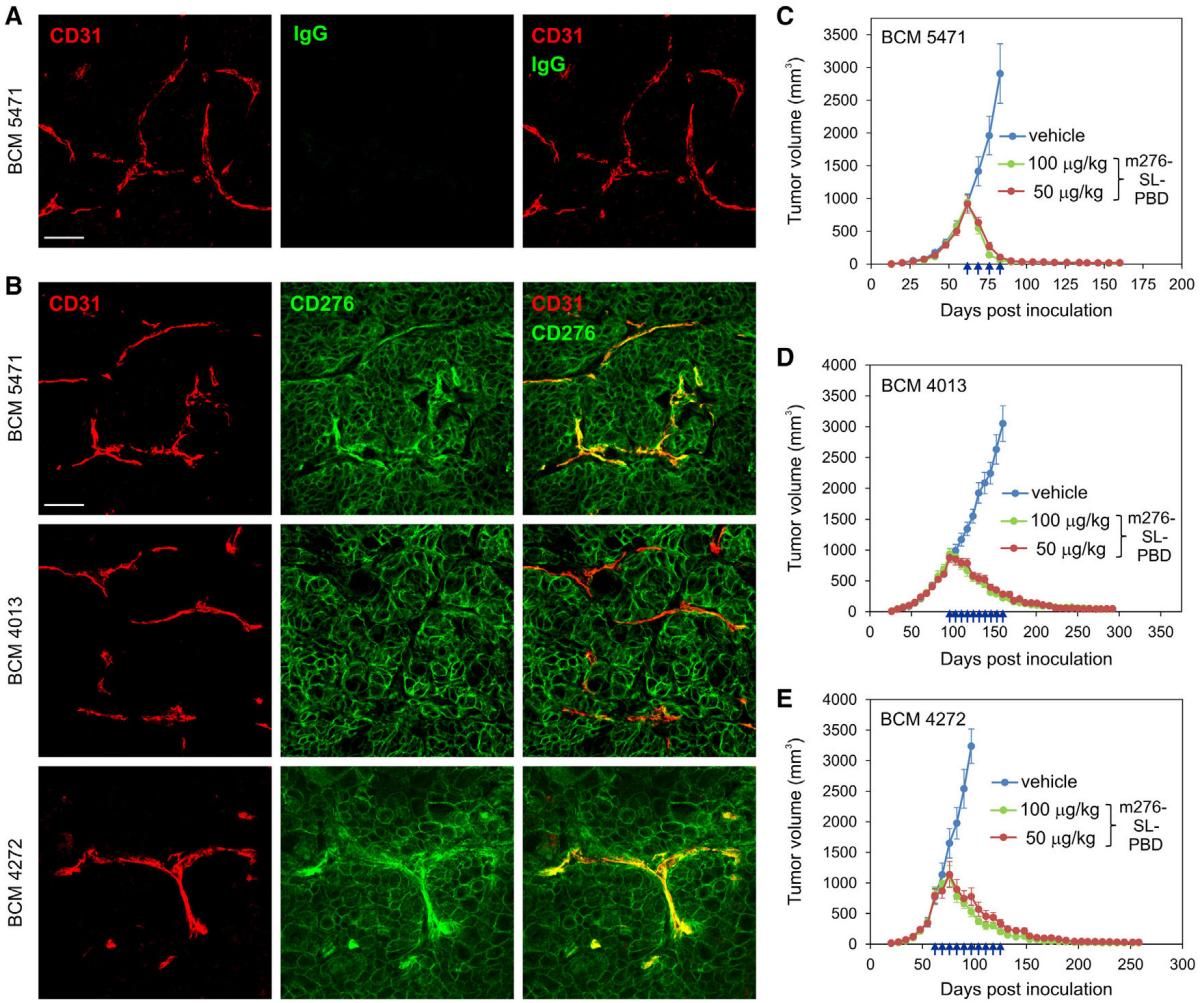
Low-dose m276-SL-PBD induces durable tumor regression in multiple CD276^+^ breast cancer PDX models (A and B) Immunofluorescence staining for CD276 (green) in BCM-5471, BCM-4013, and BCM-4272 (B) breast cancer PDX models. Tumor endothelium was co-stained with CD31 (red), confirming CD276 expression in both tumor cells and tumor vasculature. Non-specific IgG (A) served as a non-binding control. Scale bars in (A) and (B), 50 μm. (C–E) Orthotopic growth of BCM-5471 (C), BCM-4013 (D), and BCM-4272 (E) breast tumors following treatment with vehicle (control) or 50 or 100 μg/kg m276-SL-PBD. Treatments with m276-SL-PBD were initiated when tumor volumes reached ~1,000 mm^3^ and were administered on the days shown (blue arrows); n = 7–8/group. Error bars denote SEM.

**Figure 7.**
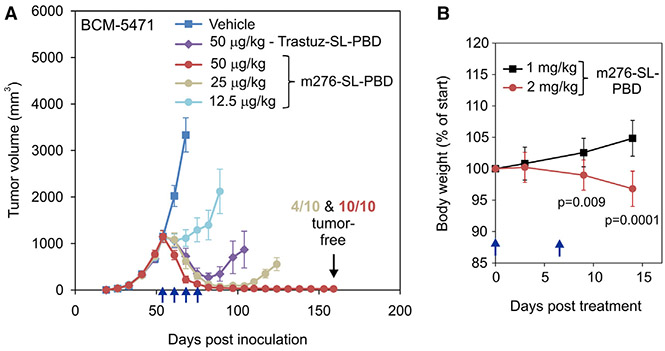
Low m276-SL-PBD doses: Well tolerated with potent anti-tumor activity (A) Orthotopic growth of BCM-5471 breast tumors in response to treatment with vehicle (control) or 12.5, 25, or 50 μg/kg m276-SL-PBD or 50 μg/kg trastuzumab-SL-PBD. ADC treatments were initiated when tumor volumes reached ~1,000 mm^3^ and were administered on the days shown (blue arrows); n = 10/group. p = 0.0003 between the 12.5 and the 25 μg/kg groups at day 89 post inoculation, and p = 0.001 between the 25 and the 50 μg/kg groups at day 124 post inoculation. Error bars denote SEM. (B) Body weights were evaluated in C57BL/6 mice following two treatments (blue arrows) with 1 or 2 mg/kg m276-SL-PBD. A t test determined p values between the two doses at the indicated time points. Error bars denote SD.

Encouraged by the sensitivity of orthotopic breast tumors derived from aggressive breast cancer cell lines, next we tested m276-SL-PBD against four CD276^+^, triple-negative orthotopic breast cancer patient-derived xenograft (PDX) models previously developed at the Baylor College of Medicine (BCM) (Figures 6A, 6B, and S7A).^52^ Immunodeficient NRG mice were initially challenged with BCM-5471 or BCM-3204 tumors. Once tumors reached an average volume of ~500–1,000 mm^3^, mice were treated with either 500 or 100 μg/kg once weekly until average tumor volumes were less than 200 mm^3^. Strikingly, complete regression was observed at both dose levels (Figures S7B and S7C). Subsequently, the ADC was tested against large 1,000-mm^3^ BCM-4013, BCM-4272,and BCM-5471 PDX models, using a dose of 100 or 50 μg/kg once per week for up to 10 weeks until the average tumor size was less than 200 mm^3^ (Figures 6C-6E and S7D-S7F). Once again, all tumors displayed a complete and durable response to the ADC treatment. To determine the minimal amount of m276-SL-PBD that was required for a complete response, mice with large BCM-5471 tumors were treated with 50, 25, and 12.5 μg/kg m276-SL-PBD once per week for 4 weeks (Figures 7A and S7G). Although these BCM-5471 tumors are classified as HER2^−^ since they have not undergone HER2 gene amplification, they still express endogenous levels of HER2 on the cell surface. For comparison, therefore, tumor-bearing mice were also treated with trastuzumab-SL-PBD. While the trastuzumab ADC, recognizing human but not mouse HER2, delayed tumor growth following treatment with 50 μg/kg ADC, all tumors eventually relapsed (Figure 7A). In contrast, for the m276-SL-PBD ADC treatment group, sustained tumor regression was observed in 4 of 10 (40%) tumors following 25 μg/kg dosing, while 100% of mice exhibited complete and sustained tumor regression following treatment with 50 μg/kg. As the MTD of m276-SL-PBD that can be administered to C57BL/6 mice without causing body weight loss is 1 mg/kg (Figure 7B), complete responses observed at the 25–100 μg/kg dose range were 10- to 40-fold lower than the MTD, indicating a wide therapeutic window.

## DISCUSSION

Over the past two decades, ADCs have gained traction as an important cancer treatment modality, with 12 clinical approvals to date. Although the concept of the ADC as a “magic bullet,” introduced by Paul Ehrlich in the early 1900s, is alluring and straightforward–combining the selectivity of an antibody with the potency of a small-molecule drug—the field has encountered numerous unexpected challenges and has achieved limited success.^53^ ADCs that are currently FDA approved generally prolong survival by only a few months compared with former standard-of-care therapy,^5,6^ and the prospect of using ADCs to obtain widespread cures remains elusive. The primary challenge in ADC development is that most, if not all, ADCs used clinically are too toxic and maintain a therapeutic index similar to that of free chemotherapeutic drugs, including the payloads used to arm them.^12,54^ Furthermore, ADC toxicity profiles often mirror those of the payload administered as a free drug and are similar among ADCs targeting various antigens but sharing the same payload.^8,12,55^ These findings raise a crucial question: if off-target toxicities are the main cause of treatment failure and the major obstacle to successful ADC development, why has it taken so long to discern them? One likely reason is that most ADCs target human antigens exclusively, and cross-reactivity with corresponding mouse antigens does not exist, making it challenging to compare on-target versus off-target toxicities in mouse models. Since most antibodies originated from murine hybridomas and were later humanized, immune tolerance mechanisms prevented cross-reactivity with mouse antigens. Because of this, as the ADC field matured, on-target off-tumor toxicities were seldom modeled in early preclinical studies but instead were usually only indirectly addressed in subsequent primate toxicity studies or phase I clinical trials. Although a few cases of “likely” on-target off-tumor toxicities have emerged, these examples are surprisingly rare.^8,56^ The m276 anti-CD276 mAb used to make the CD276 ADC described here differs from those of most ADCs in that it was selected *in vitro* using a human antibody display library. This approach, which avoids *in vivo* immune tolerance mechanisms, facilitated the identification of fully human antibodies with similar affinity toward both the mouse and the human target antigens.^14,57^ Also, because CD276 is dispensable for cell viability, this provided us with an opportunity to assess whether ADC toxicity (or efficacy) is target dependent *in vivo* using genetically engineered CD276-WT or -KO mice. Gratifyingly, using CD276-WT and -KO mice challenged with CD276 WT-or -KO cancer cells, we have been able to verify that CD276 ADC efficacy is largely target antigen dependent.^14^ However, studies with the same KO mice have helped illuminate a major limitation of our CD276 ADCs that likely also applies to many other ADCs across the field, that is, that ADC toxicities are predominantly driven by target-antigen-independent mechanisms. With this insight in mind, the major goal of this study was to systematically optimize our CD276 ADC to circumvent these off-target toxicity issues and improve the therapeutic index. We set out to thoroughly address these issues using a multipronged approach that involved identification of the most suitable payload, modification of the drug linker, enhancement of site-specific drug conjugation, Fc inactivation, optimization of the purification platform, and cancer sensitivity screening.

The choice of drug payload is a major consideration for any new ADC development program, as it can profoundly influence ADC efficacy, drug resistance, and off-target binding. Most clinically approved ADC payloads can be exported by drug efflux pumps such as P-glycoprotein, which are expressed on the CD276^+^ tumor-associated vasculature. Although we were initially drawn to PBDs due to their insensitivity to P-glycoprotein-mediated drug efflux and their ability to kill both CD276^+^ tumor vasculature and cancer cells,^14^ they also display potent bystander killing, helping to ensure killing of target-negative cells in cases where CD276 expression on tumor cells is heterogeneous. PBDs also show efficacy against both dividing and non-dividing cells, and recent studies suggest CD276 is also expressed by cancer stem cells, which are not always dividing.^58,59^ Despite these advantages, the main concern with PBDs, as with any ADC payload, is their potential for toxicity. To address this concern, we delved into understanding the underlying causes of ADC toxicity *in vivo*. Using a hydrophobic fluorophore drug surrogate, we discovered that site-specific conjugation at S239C can mitigate much of the non-specific binding caused by randomly labeling surface-exposed cysteines. The LALAPG mutations in the Fc domain are also predicted to aid in tumor targeting by eliminating unintentional binding to FcγR-expressing cells.

An unexpected discovery that emerged while testing the sensitivity of a panel of cancer cell lines, all with similar CD276 levels, was that cancer sensitivity to m276-SL-PBD depended not only on the presence of target, but also on the cancer type (Figures 4A-4F). While genetic disruption of the CD276 gene using CRISPR-Cas9 rendered sensitive cancer cell types >100-fold resistant to the m276-SL-PBD, highlighting the necessity of the target for potent killing, intrinsic factors appeared to render other cancer types relatively resistant to ADC killing even when CD276 target was present at high levels. For example, CD276^+^ neuroblastoma and breast cancers were generally much more sensitive to the ADC than CD276^+^ pancreatic cancers and glioblastomas. Its noteworthy that the same trends were observed in response to the PBD free drug itself, which can readily cross cell membranes independent of target expression levels. These results suggest that resistance is regulated by intrinsic properties of the cancer cell type of origin and/or the genetic driver mutations characterizing different cancer types. While the factors responsible for regulating PBD resistance among different cancer types require further study, potential contributors include mitotic index, apoptotic sensitivity, DNA repair capacity, drug uptake, drug trafficking, lysosomal processing, and drug metabolism or efflux.^50,51^

One challenge we faced while working with the SGD-1882 PBD payload is its extreme hydrophobicity. While this biophysical property is essential for its bystander killing activity, it also creates challenges when handling the drug *in vitro*. To address this, we introduced a hydrophilic PEG-4 group between the maleimide group and the cleavable dipeptide of the drug linker, improving its biophysical handling without adversely affecting ADC potency or toxicity *in vivo*. In addition, we observed that the conjugation of the free S239C engineered cysteines with PBD was surprisingly inefficient, and HIC purification proved effective in removing unlabeled species, resulting in a much more potent ADC.

As a stringent test of our ability to improve the therapeutic window, one of the goals of the current work was to successfully treat tumors at a size larger than that used in most preclinical studies. Large (~1,000 mm^3^) mouse tumors better reflect human tumors or their metastases, which have been estimated to have a volume of ~1,000 mm^3^ and 10^8^–10^9^ cells by the time they are detectable by clinical imaging modalities.^22,23^ Tumor size at diagnosis is the most frequently used variable to estimate prognosis; large tumors correlate with increased metastases, a high probability of developing drug resistance, and reduced survival.^24^ The development of drug resistance is directly proportional to both the mutation rate and the absolute tumor cell number,^25^ making success in treating large 1,000-mm^3^ tumors in mice potentially more predictive of future treatment success in patients. While our initial 0.5 mg/kg m276-glyco-PBD ADC was seldom able to cure mice with 1,000-mm^3^ tumors, m276-SL-PBD performed far better. By further refining the m276-SL-PBD purification strategy and selecting cancer cell types that are intrinsically more sensitive, we were able to create a fully human, fully cross-species-reactive ADC capable of eradicating large tumors at doses in the 25–50 μg/kg clinical range. It is important to note that m276-SL-PBD could be effective at even lower doses in humans, as mice typically require up to 12.3× higher doses to achieve the human equivalent dose (in μg/kg) after normalization to body surface area.^60^ In addition, several features could enable m276-SL-PBD to be tolerated at higher levels than other SGD-1882/talirine PBD-based ADCs previously tested in clinical trials. First, unlike previously tested PBD-based ADCs in the clinic, m276-SL-PBD has diminished FcγR binding, predicted to result in less non-specific uptake and killing of FcγR-positive cells of the reticuloendothelial system. Second, m276-SL-PBD has undergone an additional HIC purification step, not routinely used for PBD-ADC purification, enabling an ~3- to 4-fold increase in target-dependent killing. Third, most SGD-1882/talirine PBD-based ADCs that failed to advance through clinical trials, such as SGN-CD33A, SGN-CD70A, SGN-CD19B, SGN-CD123A, and SGN-CD352A, were targeted against hematological tumors, even though hydrophobic PBDs can readily diffuse out of target cells upon uptake and release in lysosomes.^61,62^ Finally, unlike previous SGD-1882-linked ADCs that recognized only the human target antigen in xenograft tumor studies, m276-SL-PBD cross-reacted with both human and mouse CD276 and exhibited less toxicity and increased potency and efficacy against much larger tumors.

### Limitations of the study

Several potential limitations to this study are worth mentioning. First, m276-SL-PBD has been tested only in preclinical studies, and clinical studies are needed to assess its activity in humans. Second, while we were able to remove the DAR0 species from the final ADC preparation using HIC, this additional step decreases final yields and adds additional complexity to the manufacturing process. One of the key reasons we selected S239C for site-specific labeling was to reduce surface hydrophobicity and minimize non-specific uptake *in vivo*. ADCs site-specifically conjugated to SDG-1882 at S239C have an apparently lower surface hydrophobicity, as assessed by HIC, compared with ADCs conjugated at other sites.^36^ However, while a reduction in surface hydrophobicity can potentially reduce non-specific uptake *in vivo*,^30^ low resolution during HIC separation makes DAR0 species removal challenging, necessitating a trade-off between ADC recovery (~50%) and high purity. More robust site-specific labeling methods, such as those employing unnatural amino acids,^63^ could improve homogeneity and yields, avoiding the need for HIC purification. Third, measuring the levels of free PBD released into the circulation of mice at the therapeutic doses used in these studies has been challenging due to the extreme hydrophobicity and potency of PBDs. A better understanding of drug distribution *in vivo* could aid toxicity assessments. Finally, because the functions of CD276 in normal cells, such as immune cells, remain largely unknown, the consequences of off-tumor CD276 targeting in normal cells will need careful monitoring moving forward.

To effectively target solid tumors, an ideal ADC-based therapy needs to selectively target tumors independent of anatomical location and tumor cell heterogeneity. Anti-CD276 antibodies have the potential to selectively target many solid tumors, including most, if not all, breast cancers regardless of disease subtype and stage.^14^ However, the potent activity of ADCs has, to date, been severely hindered by target-antigen-independent toxicities driven by various factors. By adopting a multipronged approach to improve the therapeutic index, these studies aim to pave the way for the development of a safer and more effective generation of ADCs, aligning with the initial goals set forth as the ADC field began.

## STAR★METHODS

### RESOURCE AVAILABILITY

#### Lead contact

Further information and requests for resources and reagents should be directed to and will be fulfilled by the lead contact, Brad St. Croix (stcroixb@nih.gov).

#### Materials availability

Reagents generated in this study, including the m276-SL-PBD ADC, will be made available upon request. Some materials may require requests to collaborators and/or agreements with various entities. Materials that can be shared will be released via a Material Transfer Agreement.

#### Data and code availability

This paper analyzes existing, publicly available data. The cancer mRNA data are available from the database: Depmap portal website: https://depmap.org/portal/, 22Q4 release. This paper does not report original code. Any additional information required to reanalyze the data reported in this paper will be shared by the lead contact upon request.

### EXPERIMENTAL MODEL AND SUBJECT DETAILS

All mice were bred and maintained in a pathogen free facility certified by the Association for Assessment and Accreditation of Laboratory Animal Care (AAALAC) International, and the study was carried out in accordance with protocols approved by the NCI Frederick Animal Care and Use Committee (ACUC). Animal care was provided in accordance with the procedures outlined in the “Guide for Care and Use of Laboratory Animals” (National Research Council; 2011; National Academies Press; Washington, D.C.). Mice were fed Charles Rivers Rat and Mouse 18% autoclavable diet (Cat # 5L79, LabDiet) *ad libitum* and maintained under conventional housing. Because all tumor studies were performed in female 10- to 16-week-old athymic NCr-nu/nu mice, the influence gender and age on the results of the study was not evaluated, potentially limiting the generalizability of the results.

### METHOD DETAILS

#### Cell lines and PDX models

The cell lines 293, MDA-MB-231, U118MG, U87MG, IMR32, ASPC1, BXPC3, HPAC, MiaPaCa2, BT549, DU4475, HCC1395, Colo205, DLD-1, RKO, SW620, A549, Calu-6, NCI-H1299 were obtained from the American Type Culture Collection. UACC-62 (UACC), HCT-116, SF295, SNB-75, SP539, U251, XF498, Pan02, Pan03, HCT-116, DMS-273, HOP-62, HOP-92, NCI-H460 were obtained from the Division of Cancer Treatment and Diagnosis (DCTD) Tumor Repository at NCI (Frederick, MD). B2-17, KP4 and YH13 were obtained from the JCRB Cell Bank, ES1 and ES4 were from the St. Jude Children’s Research Hospital, PC9 were obtained from Sigma, JIMT (JIMT-1) were obtained from AddexBio, DKMG were from the DSMZ repository, IMR5 were from Accegen Biotechnology and Cas1 were from the Interlab Cell Line Collection Catalogue (ICLC). Kelly, SK-N-DZ and SMS-SAN were a gift from Dimiter S. Dimitrov (University of Pittsburgh), D263MG and D336MG were a gift from Darell D. Bigner (Duke University), SUM159 and SUM190 were a gift from Esta Sterneck (NCI Frederick), 9464D was a gift from Paul M. Sondel (University of Wisconsin), NB-EB was a gift from Javed Khan (NIH, Bethesda), LN18 was a gift from Karlyne Reilly (NIH, Bethesda), PDA-luc4 was a gift from the Center for Advanced Preclinical Research (CAPR) at the NCI and derived from spontaneous tumors that developed in a Kras, p53, PdxCre (KPC) pancreatic tumor model. The BCM breast cancer PDX models were developed at the Baylor College of Medicine.^52^ BCM tumors were passaged as tumor fragments *in vivo* in the mammary fat pad.

#### *CD276* gene targeting in cells using CRISPR-Cas9

To create the CD276-KO vector, two DNA oligonucleotides (CD276-guide-1; 5’-CACCGTGGCACAGCTCAACCTCATC-3’ and CD276-guide-2; 5’-AAACGATGAGGTTGAGCTGTGCCAC-3’) were synthesized (Integrated DNA Technologies), phosphorylated using T4 PNK (NEB, M0201S), annealed at 95°C for 5 min, slowly cooled to RT, and ligated into a *BsmBI* (NEB #R0580)-digested lentiviral expression vector lentiCRISPR v2 (Addgene, plasmid #52961) using Quick Ligase (NEB, cat. no. M2200S). To make lentiviral particles, Lenti-X293T cells (Takara, 632180) were transfected with gRNA-encoding lentiCRISPR v2 vector along with pMD2G (Addgene, 12259) and psPAX2 (Addgene, 12260) via 3:2:5 ratio using Lipofectamine 2000 (Invitrogen, 11668019) according to the manufacturer’s manual. Lentiviral particles were then concentrated using a Lenti-X concentrator (Takara, 631232), followed by transduction into cells. 48 to 72 hr post transduction puromycin was used to select positive cells. One week after puromycin selection, expanded live cells were subjected to FACS using the rabbit anti-CD276 antibody (clone EPNCIR122, Abcam, ab134161) to isolate CD276 negative cells. CD276^+^ parent cells were used for gating and cells were sorted a second time as needed to ensure a homogenous negative population.

#### Tumor studies and body weights

For body weight analysis, ADCs or vehicle (PBS) were administered to CD276 WT and KO mice on an immunocompetent C57BL6/NCr background^14^. In these studies, CD276 WT and KO littermates derived from *Cd276*^*+/−*^ heterozygous intercrosses were randomly assigned to experimental groups. Tumor studies with HCT-116, UACC, ES4, IMR5, SUM159, MDA-MB-231, JIMT, DU4475 were performed in female 10 to 16-week-old athymic NCr-nu/nu mice (Charles Rivers) that also carried the carboxylesterase 1C (Ces1c/ES1) mutation (B6(Cg)-Ces1ctm1.1Loc/J, The Jackson Laboratory, Stock No: 014096). These humanized NCr-nu/nu mice contain Ces1c blood levels that mirror the low levels found in human plasma^65,66^. All breast tumor cell lines (DU4475, JIMT1, MDA-MB-231 and SUM159) were injected orthotopically into the mammary fat pad, while other cancer cell lines (ES4, HCT116, IMR5 and UACC) were injected subcutaneously into the flank. For PDX tumor challenge studies, fresh tumor fragments derived from live tumors passaged in mice were implanted orthotopically along with Matrigel into the inguinal mammary fat pad of 3- to 4-month-old female NRG mice (NOD.Cg-Rag1tm1Mom Il2rgtm1Wjl/SzJ, stock no. 007799, The Jackson Laboratory). Tumors were measured with a digital caliper, and tumor volumes were calculated using the formula LxW^2^x0.5 and presented as the mean ± SEM. For therapeutic studies, mice were sorted into groups containing the same average size tumors (usually 500-1000 mm^3^) prior to initiation of therapy. Mice were treated with CD276 antibodies or vehicle (PBS) at the doses and schedules described in the individual figures. During our previous ADC work we determined that i.v. and intraperitoneal (i.p.) dosing produced equivalent results^14^. Because mouse tail vein injections are difficult to perform with 100% precision and consistency, for therapeutic studies ADCs were administered by i.p. injection.

#### Metastasis and animal imaging

For experimental MDA-MB-231 breast cancer metastasis, 5x10^5^ luciferase tagged MDA-MB-231-luc cells were injected intravenously into NRG mice. 7 days after injection *In vivo* bioluminescence imaging (IVIS Spectrum imager, PerkinElmer Inc.) was used to sort mice into two groups of equal average tumor burden and i.p. treatments initiated with vehicle or 0.5 mg/mL of m276-SL-PBD once per week for four weeks. Imaging was performed on days 7, 14, 28, 42 and 180 at peak luciferin uptake, approximately 10-12 minutes after D-luciferin (30 mg/mL; 100 μL per 20 gram body weight) i.p. injection. Metastases (bioluminescence signal) were visualized using Living Image software (version 4.3.1, PerkinElmer Inc.).

#### Immunoblotting

JIMT-1 and DU4475 cell lysates were separated by SDS-PAGE and transferred to a polyvinylidene difluoride (PVDF) membrane (Millipore). Immunoblots were probed with a rat anti-CD276 monoclonal primary antibody (clone M3.2D7, ThermoScientific, #16-5973-81), followed by HRP-conjugated anti-rat secondary antibody (Jackson Immunoresearch Laboratories, #112-035-143). For HER2 detection, lysates separated by SDS-PAGE were transferred to PVDF and probed with rabbit anti- HER2/ErbB2 antibody (M45) (Cell Signaling, #3250S), followed by HRP-conjugated anti-rabbit secondary antibody (Jackson Immunoresearch, #111-035-003). CD276 and HER2 were visualized using Pierce SuperSignal West Dura Substrate (ThermoFisher Scientific, #34076) according to the supplier’s instructions, and developed using a GeneGnome ECL processor (Syngene).

#### Cell viability assays

Cell viability was measured using alamarBlue (ThermoFisher Scientific). Cells were plated in 96-well plates and 24 h later treated in triplicate with the ADCs described in the text. To minimize evaporation, plates were wrapped in cellophane during incubation. Five to 8 days after treatment, when untreated control wells were 70 to 90% confluent, 10% Alamar Blue reagent was added to the plates, and fluorescence (ex: 570 nm, Em: 585 nm) was measured on a CLARIOstar Microplate Reader (BMG Labtech) according to the manufacturer’s instructions. Wells treated identically but without cells were used to subtract background. Data was analyzed using GraphPad Prism 9.

#### Immunofluorescence staining

Tumor tissues were excised, frozen in OCT, cryosectioned, rinsed with PBS, and fixed with methanol: acetone. Sections were blocked in Tris-Buffered Saline (TBS) containing 1% blocking reagent (Roche, cat 11096176001), stained with rabbit anti-CD276 antibodies (clone EPNCIR122, Abcam ab134161), and amplified with FITC goat anti-rabbit antibodies (Jackson Immunoresearch Laboratories) followed by Alexa 488 donkey anti-goat antibodies (Jackson Immunoresearch Laboratories). Endothelial cells were co-localized using rat anti-CD31 antibodies (clone MEC13.3, Santa Cruz) followed by biotin-labeled donkey anti-rat antibodies (Jackson) and Texas red-streptavidin (Vector laboratories). Nuclei were counterstained with DAPI (Hoechst 33258, ThermoFisher cat# H3569) prior to mounting with DAKO Fluorescent mounting medium (cat# S3023). Specificity of staining was verified by substituting primary antibodies with isotype matched non-specific IgGs. Images were captured with a Zeiss LSM 780 confocal microscope and analyzed using Zen 3.2 software.

#### Immunohistochemistry of PDX samples

Formalin-fixed paraffin embedded (FFPE) sections were deparaffinized, treated with Dual Endogenous Enzyme-Blocking Reagent (Dako) followed by the Biotin blocking system (Dako) and blocked with 1% blocking reagent (Roche) in TBS (100 mM Tris (pH 7.5), 150 mM NaCl + 1% Triton X 100). Sections were incubated with goat anti-human CD276 (R&D catalogue # AF1027) for 2 hr at room temperature followed by signal amplification using a Vectastain ABC HRP Kit (Vector Laboratories). The goat anti-human CD276 antibody from R&D used for IHC staining of human PDX tumor tissues was also found to react specifically with murine CD276 in tumor vessels of CD276 WT but not KO mice^14^.

#### Flow cytometry

Pilot studies revealed that CD276 on the surface of cultured cells is insensitive to brief trypsinization. Therefore, cells were trypsinized, rinsed in cold PBS/0.5%BSA (PBS/BSA), and labeled with m276, m276-SL or isotype control human anti-CD276 mAb in PBS/BSA at 4°C. Cells were rinsed with PBS/BSA and incubated with FITC-conjugated donkey anti-human IgG secondary antibodies (Jackson Immunoresearch, #709-095-149), West Grove, PA), rinsed again, and analyzed on a LSRFortessa (Becton Dickinson). All flow cytometry data were analyzed using FlowJo software (FlowJo, LLC).

#### Analysis of CD276 RNA across cancer cell lines

The normalized RNA expression level (log2(TPM+1)) of CD276 across certain cancer cell types (B-Lymphoblastic Leukemia, Glioblastoma, Neuroblastoma, Pancreatic Cancer) was downloaded from the DepMap (https://depmap.org/portal/, 22Q4 release). Data were analyzed and plotted using R software (4.2.1).

### ELISA

To compare m276-SL and m276SL-PBD binding to CD276, human CD276 ECD protein was coated onto a high-binding ELISA plate (Santa Cruz, #sc-204463) at 100 ng/well overnight at 4°C. The next day, the wells were blocked with MP buffer (2% Milk/PBS) for 1 h at 37°C. The test antibodies, m276-SLand m276-SL-PBD, were serially diluted 1:5 in MP buffer and added to wells in duplicates and incubated for 2 h at 37°C. The detection antibody was goat anti-human (H+L)-HRP (Jackson Immunoresearch) diluted 1:2000 in MP buffer. After 1 h at 37°C incubation, the plate was washed with 0.05% Tween 20 in PBS. To develop the signals, HRP substrate ABTS (Roche, #11684302001) was added, and plates read for absorbance at 405 nm within 5 minutes on a CLARIOstar Microplate Reader (BMG Labtech). Isotype matched human IgG1 control antibody was included as a non-binding control.

#### ADC production and purification

A fully human m276-SL expression plasmid containing S239C, L234A, L235A, and P329G (LALAPG) in the Fc domain of IgG1 was used to create a stable CHOK1 m276-SL expression cell line. For large scale production, stable cells were grown in Dynamis medium (Gibco, A2661501) with 25 μM MSX in fed-batch culture. The culture supernatant was collected on Day 12 or when the viability dropped below 60%, whichever occurred first. Antibody in the supernatant was purified using protein G affinity chromatography and dialyzed into PBS, pH 7.4. The purity of the antibody was monitored using SDS-PAGE and SEC-HPLC and antibodies with <5% aggregates were used for further ADC production. The DS-7300a ADC was generated by producing and purifying the B7-H3 humanized M30 antibody [Sequence 85 (HC) and 77 (LC) derived from US patent 9,371,395 B2] and randomly conjugating it to the exatecan derivative-based cytotoxic payload DXd (MedChemExpress, cat # HY-13631D) on endogenous cysteines via a cleavable tetrapeptide to create an average DAR of 4 as previously described.^20^ m276-Tesirine was generated by randomly conjugating the m276 parent antibody to the drug-linker Tesirine (MedChemExpress) to obtain an average DAR of 2 as previously described.^67^

#### PBD conjugation to m276-SL via S239C

m276-SL in 1 mM EDTA/PBS was treated with a 40-molar excess of freshly made TCEP (37°C x 1 h) to reduce the interchain disulfide bonds and decap the cysteine-239. At the end of the reaction, unused TCEP was removed by dialysis into EDTA/PBS. The interchain disulfide bonds were re-oxidized by a 30-molar excess of dehydroascorbic acid for 4 h at room temperature. This partial oxidation left the free cysteine-239 in the reduced state for drug conjugation. Next, ma-(peg)_x_-VA-PBD (SGD-1882) drug-linker at >97% purity (Levena Biopharma or Creative Biolabs), was dissolved in the solvent propylene glycol (PPG) and then added to the antibody solution at 4:1 drug-linker:Ab molar ratio. The final mixture was adjusted to 50% PPG/PBS to keep both antibody and payload soluble. The reaction was incubated for 2 h at RT, then overnight at 4°C. Payload ma-(peg)_x_-VA-PBD, with x=0, 2, 4, or 8, was used for the study. While x=0 payload (PEG0) drug-linker was only partially soluble in PPG, with particulates remaining even after incubation at 55°C, all other payloads dissolved readily in PPG. After the reaction, L-cysteine was added in 3-fold molar excess of the drug-linker to quench unreacted PBD drug-linker and increase its hydrophilicity for improved removal later. Afterwards, SEC was used to monitor ADC homogeneity (A280) and payload labeling (A330). The original m276, which lacks the S239C mutation, was subjected to the same conjugation procedure, but did not have detectible PBD molecules conjugated verifying site-specific conjugation at S239C.

#### m276-SL-PBD DAR determination

The m276-SL-PBD DAR was determined by two methods: intact MS and RP-HPLC. LC-MS analysis was carried out using a Waters XEVO G2S TOF mass spectrometer and a POROSHELL 300SB C3 column (2.1 x 12.5 mm, 5 μm) connected to a Waters Acquity H Class UPLC system. The mobile phase was buffer A (0.1% formic acid in water). A gradient (2.5 min 10% B, 10-80% B gradient in 3.5 min) was applied using Buffer B (acetonitrile, 0.1% formic acid) at a flow rate of 0.4 mL/min. 10 μL of ADC solution diluted to 0.5 mg/mL with endotoxin free water was injected for analysis. Average DAR was calculated by weighted average of main peak signal intensity (SI) for each DAR species (i.e., from DAR 0 to DAR 3) on the deconvoluted mass spectra using the formula: Average DAR=∑ni=0Sli×i∑ni=0Sli. For DAR determination by RP-HPLC, 25 μg of naked m276 antibody (unlabeled control) or ADC was diluted into 50 μl PBS, reduced with 10 mM DTT and NaBH_2_CN at 37°C for 30 min, then immediately injected (3 μl) on an Agilent PLRP-S reverse phase column (2.1x50 mm, 5 um, 1000A) using an Agilent 1260 Infinite II HPLC system. The column was equilibrated with Mobile phase A (0.1% TFA in water) and maintained at 80°C. The separation of LC, HC and HC conjugated with PBD was achieved using a flow rate of 0.8 ml/min and a linear gradient of 0-100% mobile phase B (0.05% TFA in acetonitrile) for 15 min. The photo diode array (PDA) was set to detect A280 (protein) and A330 nm (PBD). HC+PBD peaks were identified based on the retention time relative to the naked HC peak, as well as the A330 values. DAR was determined using the integrated area under peak from the chromatograms.

#### Preparative hydrophobic-interaction chromatography (HIC)

Because site specific conjugation at S239C eliminates much of the surface hydrophobicity caused by the PBD drug linker^36^, the HIC protocol used for purification was optimized in order to maximize its resolving power. An XK26/20 column packed with ToyolPearl Phenyl 650S resin (Tosoh Bioscience) was equilibrated with buffer A (50 mM NaH_2_PO_4_, pH 7.0, 2 M NaCl). The ADC sample was diluted 1:1 with loading buffer (50 mM NaH_2_PO_4_, pH 7.0, 4 M NaCl). The sample was eluted with 0 to 100% Buffer B (50 mM NaH_2_PO_4_, pH 7.0, 20% isopropanol) in 20 column volumes at a flow rate of 5.5 mL/min. LSMS was used to verify that the early elution peak contained unconjugated antibody while the late peak contained the enriched DAR2 ADC. To remove residual payload, the DAR2 enriched sample was incubated with activated charcoal at RT for 1 hr. Approximately 16 μL (10% w/v charcoal in PBS) charcoal was used for 1 mg ADC. To separate the ADC solution from the charcoal slurry the mixture was centrifuged at 5000g x 10 min, and supernatant was filtered through 0.2 μm filter. The ADC was buffer exchanged into PBS, concentrated and filtered again with a 0.2 μm filter.

#### SEC to remove aggregates

All antibody samples before and after conjugation were monitored for the percentage of aggregates with a Cytiva Superdex200 Increase 10/300GL column equilibrated with PBS. The column was calibrated with molecular weight standards of 600, 400, 158, 75 and 43 kDa. In cases where the plain m276-SL had aggregates >5%, the pre-conjugation material was purified with HiLoad 26/600 Superdex200 PG column equilibrated with PBS.

#### Pharmacokinetic studies

7–8-week-old C57BL/6Ncr mice (Charles River Laboratories, Inc) were sorted randomly into two groups and treated with a single intravenous dose of either m276-SL or m276-SL-PBD (2.5mg/kg). Baseline serum was collected from mice before injection. Following injection serum was collected at the following times: 5 min, 30 min, 2 h, 4 h, 8 h, 24 h, 48 h, 72 h, 96 h, day 7, day 10, and day 14. Sera from three mice per time point were collected and stored at −80°C until the time of analysis. The concentration of m276-SL or m276-SL-PBD was measured with a verified ELISA: biotin labeled human CD276 was coated on an MSD streptavidin narrow well plate. Diluted serum was added to the wells in duplicate. The bound antibody was detected with anti-human Ab coupled with sulfo-tag. The final signal was read using Mesoscale Discovery equipment and the PK data analyzed using PK Solution 2.0 software (Summit Research Services in Montrose CO).

#### Q-TOF LC/MS analysis

Mass spectrometry data were acquired on an Agilent 6520 Accurate-Mass Q-TOF LC/MS System, (Agilent Technologies, Inc., Santa Clara, CA) equipped with a dual electro-spray source, operated in the positive-ion mode. Separation was performed on Zorbax 300SB-C3 Poroshell column (2.1 mm x 75 mm; particle size 5 μm). The analytes were eluted at a flow rate of 1 ml/min with a 5 to 90% organic gradient over 5 minutes and holding organic for 1 minute. Both mobile phases, water and acetonitrile, contained 0.1% formic acid. The instrument was used in a full-scan TOF mode. MS source parameters were set with a capillary voltage of 4 kV, the fragmentor voltage of 220 V and skimmer 65 V. The gas temperature was 350 °C, drying gas flow 12 l/min and nebulizer pressure 55 psig. Data were acquired at high resolution (3,200 m/z), 4 GHz. TOF-MS mass spectra were recorded across the range 100–3,200 m/z. To maintain mass accuracy during the run time, an internal mass calibration sample was infused continuously during the LC/MS runs. Data acquisition was performed using Mass Hunter Workstation (version B.06.01). For data analysis and deconvolution of mass spectra Mass Hunter Qualitative Analysis software (version B.07.00) with Bioconfirm Workflow was used.

#### Preparation of AFCs - Cy7 labeling on endogenous cysteines

In a 1.5 mL microcentrifuge tube, 1 mg/mL of m276 antibody in PBS with 1 mM EDTA was prepared. To conjugate Lumiprobe Cy7 maleimide to antibody, endogenous cysteines were reduced with a 40-fold molar excess of TCEP, mixed quickly by inversion, then stirred at 37 °C in the dark for 1 h. After removing excess TCEP by extensive dialysis in PBS overnight at 4 °C (Slide-A Lyzer, 10K MWCO, ThermoFisher), the antibody solution was transferred to a 1.5 mL microcentrifuge tube and mixed with a 10-fold molar excess of Lumiprobe Cy7 maleimide (6.66 μL of 10 mM DMSO stock solution) and gently shaken at room temperature (RT) in the dark overnight. Unreacted free dye was then removed from the reaction mixture using a Zeba spin DS column (7K MWCO, Thermo Fisher Scientific) equilibrated to pH 7.4 PBS. The antibody-dye conjugate mixture was again extensively dialyzed in PBS overnight at RT using a 10K MWCO Slide-A Lyzer and then concentrated to 500 μL using centrifugal filter (3 kDa cutoff). For Cy7 labeling on S239C a partial oxidation was performed using 100-fold molar excess dehydroascorbic acid before Lumiprobe Cy7 maleimide was added. The labelled antibody was further purified through a Superdex 200 Increase 10/300 GL size-exclusion column and eluted with PBS. Antibody-dye conjugates were used for animal studies within a week of labeling.

#### AFC DAR determination

The absorbance spectra of m276-hinge or m276-S or m276-SL-Cy7 dye conjugates were collected in PBS buffer (pH 7.2,1X). The drug to antibody ratio (DAR) was calculated using the following equation:

$$\text{DAR}=\frac{A_{dye}\epsilon_{m276}}{(A_{280}-A_{dye}C_{280})\epsilon_{dye}}$$

where $A_{\text{dye}}$ is the maximum monomeric absorbance of lumiprobe Cy7 maleimide, $\epsilon_{m276}$ is the molar extinction coefficient of m276 at 280 nm, which equals to 200,000 M^−1^ cm^−1^, $A_{280}$ is the absorbance of the conjugate at 280 nm, $\epsilon_{\text{dye}}$ is the molar extinction coefficient of the dye, $C_{280}$ is the correction factor of the dye at 280 nm, which is determined by $A_{280}∕A_{\lambda \max}$ of a free dye absorbance.

**Table:** 

	m276-hinge-Cy7	m276-SL-Cy7	m276-S-Cy7
$C_{280}$	0.05	0.05	0.05
$A_{\text{dye}}$	0.211	0.152	0.205
$A_{280}$	0.109	0.152	0.129
$\varepsilon_{\text{dye}}$	122500	122500	122500
DAR	3.5	1.72	2.82

While the Cy7 labeling was slightly higher in the randomly labelled hinge group compared to the site-specific groups in the experiment shown in Figure 2A, similar *in vivo* imaging results were obtained when the labeling efficiency was slightly skewed towards site-specifically labelled ADCs, indicating that the increased DAR in the hinge group was unlikely responsible for the altered biodistribution *in vivo*.

### *In vivo* immunofluorescence imaging

5x10^6^ human breast cancer cells (JIMT-1) in 100 μL of Matrigel: PBS (1:1) were injected into the inguinal mammary fat pad of 4-7-week-old female athymic nude (Charles River Laboratories International, Inc. Frederick, MD). *In vivo* imaging studies were initiated 21 days later when tumors reached 200-250 mm^3^ in size. Fluorescence images were acquired on an IVIS spectrum imager (PerkinElmer Inc.). Mice body temperature was maintained at ~37 °C during the imaging procedure with a heated pad located under the anesthesia induction chamber, imaging table, and post-procedure recovery cage. All mice were anesthetized in the induction chamber with 3% isoflurane with filtered (0.2 μm) air at 1 liter/minute flow rate for 3-4 minutes and then modified for imaging to 2% with O_2_ as a carrier with a flow rate of 1 liter/minute. Static 2D images were acquired using an excitation filter: 745 ± 15 nm, and emission filter 800 ± 10 nm, f/stop:2, Binning (8x8) and exposure: auto (typically 1-120 seconds).

Living Image software (version 4.5.5, PerkinElmer Inc.) was used for image analysis. Using white light images, the tumors were identified, and regions of interest (ROI) were drawn over the tumor, liver, and neck (used as background) to quantify *in vivo* fluorescence signals. Fitted ROIs were drawn over each organ to quantify *ex vivo* data. Total radiance efficiency within each ROI was normalized by the corresponding area of the ROI. This normalized radiance efficiency was used to calculate ratios (tumor-to-background, tumor-to-liver, and liver-to-background). Statistics (unpaired 2-tail Student’s t-test) were carried out using Prism 9.

### Synthesis of the DFO conjugates and Zirconium-89 radiolabeling

DFO conjugates (DFO-m276-SL and DFO-m276-SL-PBD) were prepared as previously described using a 5-fold molar excess of p-isothiocyanatobenzyl-desferrioxamine (DFO-Bz-NCS) (Macrocyclics, Inc.). The concentrations of the conjugates were measured using bicinchoninic acid assay (BCA) (Thermo Fisher Scientific). Zirconium-89 labeled conjugates ([^89^Zr]Zr-DFO-m276-SL and [^89^Zr] Zr-DFO-m276-SL-PBD) were also prepared according to previously described methods with minor modifications^68^. Briefly, a stock solution of zirconium-89 oxalate (~440 MBq) (3D Imaging) was diluted with 300 μL of HEPES buffer (0.5 M, pH 7.1 −7.3). From this stock solution, ~15 MBq of zirconium-89 was used per radiolabeling reaction. To the aliquot of zirconium-89 (~15 MBq, 30 μL) was added 2,5-Dihydroxybenzoic acid (20 μL, ~5 mg/mL, pH adjusted to 7 with 2M Na_2_CO_3_ solution) followed by a HEPES buffer solution of DFO-m276-SL or DFO-m276-SL-PBD (~400 μg, ~300 μL). The reaction mixture, pH 7.3-8.0, was incubated for 1 h at RT and challenged with DTPA (5 μL, 0.1 M, pH 7) for an additional 10 min. The radiolabeled conjugates were purified by a PD-10 desalting column (GE Healthcare Biosciences) using 0.9% NaCl (pH 7). The molar activity and the purity of the radiolabeled conjugates were determined by analytical high-performance liquid chromatography (HPLC) (Agilent 1200 Series instrument) using a size exclusion column $\left(t_{R}=8.0\;\text{min}\right)$ (TSKgel SuperSW3000, Tosoh Bioscience LLC). The isolated radiochemical yields were in the range of 90-95% (n = 6) with radiochemical purity >95%. The molar activities of the radio conjugates were 4800-5550 MBq/μmol (n = 6).

### PET/CT

PET-imaging studies were performed on CD276-WT C57BL/6 mice (n = 5 females) bearing 9464D-CD276-KO or 9464D-CD276-WT tumors on the left and right flanks, respectively. 3.98 ± 0.45 MBq of radiolabeled naked antibody ([^89^Zr]Zr-DFO-m276-SL-DFO) or 4.38 ± 0.36 MBq of radiolabeled ADC ([^89^Zr]Zr-DFO-m276-SL-PBD-DFO) were formulated in 200 μL of 0.9% saline and administered (i.v. tail-vein injection) to each mouse. Mice were then imaged by positron emission tomography/computed tomography (PET/CT) at approximately 4, 24, 48, 76, 120, and 168 hours post injection (nanoScan SPECT/CT/PET, Mediso, Hungary). Animals remained conscious and were allowed free access to food and water prior to and after radiopharmaceutical injection. Body temperature was maintained before and during imaging using a thermostat controlled circulating warm air imaging table. The pulmonary function was monitored during scanning and the anesthesia (1.5–2% isoflurane in O_2_ at 1 L/min) was regulated to maintain a pulmonary rate between 50 and 90 breaths per minute. Mice were imaged in the prone position fora 4 min CT for PET attenuation correction, followed by a 20 min PET acquisition. CT acquisition parameters were: 50 kVp, 980 μA, 300 ms per step, 360 steps covering 360°. PET list-mode data were acquired using an energy window of 400–600 keV and a 5 ns coincidence timing window. CT images were reconstructed using a cone beam algorithm resulting in a 0.13 mm voxel and PET utilized Ordered Subset Expectation Maximization (OSEM-3D) with 4 subsets and 4 iterations resulting in a 0.4 mm voxel. PET images were corrected for attenuation, scatter, radioactive decay and deadtime. PET/CT DICOM (radiologic format) images were displayed and fused on a MIM workstation (v 7.0.5, MIM Software Inc, Cleveland, OH). Tumor or liver [^89^Zr]-conjugate uptake was registered using a volume of interest (VOI) defined by CT, and the maximum standardized uptake value normalized by body weight (SUVbw max) was calculated using the same commercial software.

### Biodistribution

Biodistribution studies were performed on CD276 WT or KO C57BL/6 mice (n = 6 female mice per group) with 9464D-CD276-KO or 9464D-CD276-WT tumors on the left and right flanks, respectively, or in CD276 WT athymic nude mice (n = 6 female mice per group) with human ES4-CD276-KO and ES4-CD276-WT tumors on the left and right flanks, respectively. 0.444 ± 0.043 MBq of radiolabeled [^89^Zr]Zr-DFO-m276-SL parent antibody or 0.452 ± 0.06 MBq of radiolabeled [^89^Zr]Zr-DFO-m276-SL-PBD ADC were formulated in 200 μL of 0.9% saline and administered (i.v. tail-vein injection) to each mouse. Mice were then euthanized (CO_2_ followed by cardiac exsanguination) at 48 h post-injection (maximum uptake as determined in the PET/CT study). Organs were excised, weighed, and counted in the gamma well counter (Wallac Wizard 1480 3”, Perkin Elmer, Waltham, MA): blood (10 μL), tumor (left flank), tumor (right flank), liver, lung, muscle, heart, bone (femur), kidney, spleen, stomach, small and large intestines, brain, skin, axial lymph nodes, tail, and remaining carcass. The samples were counted for 1 min in the gamma counter using an energy window of 800–1,000 keV for ^89^Zr (gamma-ray energy 909 keV; T_1/2_ = 78.4 hr). Dose calibrators are not accurate at these low activities (~0.37 MBq), therefore, 10 μL from the injection vial was obtained at the time of injection. The injection syringe was weighed pre and post injection. The 10 μL injection calibration sample was measured in the gamma well counter at the same time as the organs and the resultant measurement (counts per minute: CPM) is corrected for dead-time for conversion to disintegrations per minute (DPM), converted to a unit volume (DPM/μL), multiplied by the net syringe volume (μL), subtracted by the activity in the tail, and decayed to the injection time to obtain the net injected activity (MBq) per mouse. Organ gamma-counter measurements were also corrected for dead-time, background, and normalized by injected dose and organ weight to obtain percent injected activity per gram of tissue (%ID/g). Calculation of the %ID/g of blood was also corrected for dead-time, background, and injected activity. Total blood volume was converted to weight by the blood density (1.06 g/mL) and the average rodent blood volume (78 mL/kg).^69^

### QUANTIFICATION AND STATISTICAL ANALYSIS

A Students t-test was used to calculate differences in tumor volumes, body weights or differences in lumiprobe Cy7 fluorescence between two groups. An ANOVA was used calculate differences between three or more groups with Tukey’s post-hoc method to control for experiment-wise error. For Kaplan Meier survival analysis, a Log-rank (Mantel-Cox) test was used to compare each of the arms. All measurements were taken from distinct samples. Differences between two groups were presented as the mean ± s.e.m. or mean ± s.d. as noted in Figure Legends. Experimental sample numbers (n) are indicated in Figure Legends. All tests were two-sided and p values < 0.05 were considered statistically significant. All statistical analysis was performed with GraphPad Prism 9.4.1.

## Supplementary Material

1

## Figures and Tables

**Table:** KEY RESOURCES TABLE

REAGENT or RESOURCE	SOURCE	IDENTIFIER
Antibodies		
m276	(Seaman et al., 2017)^14^	N/A
m276-S	This paper	N/A
m276-SL	This paper	N/A
m276-SL-PBD	This paper	N/A
^89^Zr-m276-SL	This paper	N/A
^89^Zr-m276-SL-PBD	This paper	N/A
m276-tesirine	This paper	N/A
m276-hinge-Cy7	This paper	N/A
m276-S-Cy7	This paper	N/A
m276-SL-Cy7	This paper	N/A
DS-7300a	This paper	N/A
Rabbit Anti-CD276 (clone: EPNCIR122)	Abcam	Cat# ab134161; RRID:AB_2687929
Peroxidase AffiniPure Goat Anti-Rat IgG (H+L)	Jackson ImmunoResearch	Cat# 112-035-143; RRID:AB_2338138
Peroxidase AffiniPure Goat Anti-Rabbit IgG (H+L)	Jackson ImmunoResearch	Cat# 111-035-003; RRID: AB_2313567
FITC Goat Anti-Rabbit IgG (H+L)	Jackson ImmunoResearch	Cat# 111-095-144; RRID: AB_2337978
Biotin-SP F(ab’)_2_ Fragment Donkey Anti-Rat IgG (H+L)	Jackson ImmunoResearch	Cat# 712-066-150; RRID: AB_2340648
Fluorescein (FITC) AffiniPure Donkey Anti-Human IgG (H+L)	Jackson ImmunoResearch	Cat# 709-095-149; RRID: AB_2340514
Goat Anti-Human B7-H3	R&D Systems	Cat# AF1027; RRID: AB_354546
Rat Anti-Mouse PECAM-1 (clone: MEC 13.3)	Santa Cruz Biotechnology	Cat# sc-18916; RRID: AB_627028
CD276 (B7-H3) Monoclonal Antibody (M3.2D7)	Thermo Fisher	Cat# 16-5973-81; RRID:AB_469172
Donkey Anti-Goat IgG (H+L), Alexa Fluor 488	Thermo Fisher	Cat# A11055; RRID: AB_142672
HER2/ErbB2 (M45) Antibody	Cell Signaling Technology	Cat# 3250; RRID:AB_2231241
Chemicals, peptides, and recombinant proteins		
DAKO Fluorescence Mounting Medium	Agilent Technologies Inc	Cat# S3023
HiLoad 26/600 Superdex 200 pg	Cytiva	28989336
D-Luciferin, Potassium Salt	Gold Biotechnology	Cat# LUCK-4G
Cy7 maleimide	Lumiprobe Life Science Solutions	25080
T4 PNK	New England Biolabs	M0201S
BsmBI	New England Biolabs	R0580
Quick Ligase	New England Biolabs	M2200S
Blocking Reagent	Roche	Cat# 11096176001
ABTS^™^ Solution	Sigma Aldrich	11684302001
Lenti-X 293T	Takara	632180
Lenti-X concentrator	Takara	631232
Hoechst 33258, Pentahydrate (bis-Benzimide)	ThermoFisher	Cat# H3569
Dynamis^™^ Medium	ThermoFisher	A2661501
TOYOPEARL Phenyl-650S	TOSOH BIOSCIENCE LLC	14477
Texas Red Streptavidin	Vector Labs	Cat# SA-5006; RRID: AB_2336754
Dako Dual Endogenous Enzyme-Blocking Reagent	Agilent Technologies Inc	Cat# S200380-2
Dako Biotin Blocking System	Agilent Technologies Inc	Cat# X059030-2
SuperSignal^™^ West Dura Extended Duration Substrate	Thermo Fisher	Cat# 34076
alamarBlue^™^ Cell Viability Reagent	Thermo Fisher	Cat# DAL1100
Vectastain Elite ABC Kit (goat IgG)	Vector Labs	Cat# PK-6105; RRID: AB_2336824
DAKO Fluorescence Mounting Medium	Agilent Technologies Inc	Cat# S3023
HiLoad 26/600 Superdex 200 pg	Cytiva	28989336
D-Luciferin, Potassium Salt	Gold Biotechnology	Cat# LUCK-4G
Cy7 maleimide	Lumiprobe Life Science Solutions	25080
ma-(peg)_x_-VA-PBD (SGD-1882)	Levena Biopharma	MA-(PEG)x-VA-PBD
ma-(peg)_4_-VA-PBD (SGD-1882)	Creative Biolabs	MA-PEG4-VA-PBD (ADC-S-043)
Tesirine	MedChemExpress	HY-128952
Dxd	MedChemExpress	HY-13631D
T4 PNK	New England Biolabs	M0201S
BsmBI	New England Biolabs	R0580
Quick Ligase	New England Biolabs	M2200S
Blocking Reagent	Roche	Cat# 11096176001
ABTS^™^ Solution	Sigma Aldrich	11684302001
Lenti-X 293T	Takara	632180
Lenti-X concentrator	Takara	631232
Hoechst 33258, Pentahydrate (bis-Benzimide)	ThermoFisher	Cat# H3569
Dynamis^™^ Medium	ThermoFisher	A2661501
TOYOPEARL Phenyl-650S	TOSOH BIOSCIENCE LLC	14477
Texas Red Streptavidin	Vector Labs	Cat# SA-5006; RRID: AB_2336754
Critical commercial assays							
Dako Dual Endogenous Enzyme-Blocking Reagent	Agilent Technologies Inc	Cat# S200380-2
Dako Biotin Blocking System	Agilent Technologies Inc	Cat# X059030-2
SuperSignal^™^ West Dura Extended Duration Substrate	Thermo Fisher	Cat# 34076
alamarBlue^™^ Cell Viability Reagent	Thermo Fisher	Cat# DAL1100
Vectastain Elite ABC Kit (goat IgG)	Vector Labs	Cat# PK-6105; RRID: AB_2336824
Deposited data		
Gene expression data from DepMap portal	DepMap	https://depmap.org/portal/, 22Q4 release
Experimental models: Cell lines		
IMR-5	Accegen Biotechnology	ABC-TC0450
JIMT-1	AddexBio	C0006005
HEK293	ATCC	CRL-1573; RRID: CVCL_0045
IMR-32	ATCC	CCL-127; RRID:CVCL_0346
AsPC-1	ATCC	CRL-1682; RRID:CVCL_0152
BxPC-3	ATCC	CRL-1687; RRID:CVCL_0186
HPAC	ATCC	CRL-2119; RRID:CVCL_3517
DU4475	ATCC	HTB-123; RRID:CVCL_1183
HCC1395	ATCC	CRL-2324; RRID:CVCL_1249
SW620	ATCC	CCL-227; RRID:CVCL_0547
NCI-H1299	ATCC	CRL-5803; RRID:CVCL_0060
BT549	ATCC	N/A
PDA luc4	CAPR	N/A
D263-MG	Darell D. Bigner, Duke	N/A
D336-MG	Darell D. Bigner, Duke	N/A
MDA-MB-231	DCTD	N/A
HCT 116	DCTD	N/A
SF295	DCTD	N/A
SNB-75	DCTD	N/A
U251	DCTD	N/A
PAN02	DCTD	N/A
PAN03	DCTD	N/A
HCT 116	DCTD	N/A
DMS-273	DCTD	N/A
HOP-62	DCTD	N/A
HOP-92	DCTD	N/A
NCI-H460	DCTD	N/A
COLO 205	DCTD	N/A
A549	DCTD	N/A
Kelly	Dimiter S. Dimitrov	N/A
SK-N-DZ	Dimiter S. Dimitrov	N/A
SMA-SAN	Dimiter S. Dimitrov	N/A
DK-MG	DSMZ	ACC 277; RRID:CVCL_1173
SUM159	Esta Sterneck	N/A
SUM190	Esta Sterneck	N/A
MIA PaCa-2	ATCC	CRM-CRL-1420; RRID:CVCL_0428
DLD-1	ATCC	CCL-221; RRID:CVCL_0248
CAS-1	ICLC	HTL97009; RRID:CVCL_1117
NB-EB	Javed Khan	N/A
B2-17	JCRB Cell Bank	IFO50361; RRID:CVCL_2864
KP-4	JCRB Cell Bank	JCRB0182; RRID:CVCL_1338
YH-13	JCRB Cell Bank	IFO50493; RRID:CVCL_1795
U87MG	Karlyne Reilly	N/A
LN18	Karlyne Reilly	N/A
RKO	Long Dang	N/A
9464D	Paul M. Sondel	N/A
PC9	Sigma Aldrich	90071810; RRID:CVCL_1640
ES1	St. Jude Children’s Research Hospital	N/A
ES4	St. Jude Children’s Research Hospital	N/A
U118MG	ATCC	HTB-15; RRID:CVCL_0633
Calu-6	ATCC	HTB-56; RRID:CVCL_0236
UACC-62	DCTD	N/A
SF539	DCTD	N/A
XF498	DCTD	N/A
Experimental models: Organisms/strains		
Cd276^−/−^ mouse	(Seaman et al., 2017)^14^	N/A
NCr-nu/nu	Charles Rivers	NCr-nu/nu
B6(Cg)-Ces1ctm1.1Loc/J	The Jackson Laboratory	14096; RRID:IMSR_JAX:014096
NOD.Cg-Rag1tm1Mom Il2rg/SzJ	The Jackson Laboratory	RRID:IMSR_JAX:007799
C57BL/6NCrl	Charles River	RRID:IMSR_CRL:027
Oligonucleotides		
CD276-guide-1; CACCGTGGCACAGCTCAACCTCATC	This paper	Custom synthesis IDT
CD276-guide-2; AAACGATGAGGTTGAGCTGTGCCAC	This paper	Custom synthesis IDT
Recombinant DNA		
lentiCRISPR v2	(Sanjana et al.)^64^	Addgene 52961; RRID: Addgene_52961
pMD2G	unpublished	Addgene 12259; RRID: Addgene_12259
psPAX2	unpublished	Addgene 12260: RRID: Addgene_12260
Software and algorithms		
PK Solution 2.0 software	Summit Research Services	www.pharmpk.com
Mass Hunter Qualitative Analysis software,version B.07.00	Agilent	www.agilent.com
Living Image, version 4.3.1	PerkinElmer	www.perkinelmer.com
FlowJo software, version 10.8.1	FlowJo, LCC	www.flowjo.com
GraphPad Prism 9.4.1	GraphPad, LCC	www.graphpad.com
MIM workstation, version 7.0.5	MIM Software Inc.	www.mimsoftware.com/
R software, version 4.2.1	R software	https://cran.r-project.org/

## Highlights

- CD276 is an effective target for next-generation ADC therapy
- CD276 ADCs can eradicate large 1,000-mm^3^ tumors in preclinical trials
- Genetic and chemical engineering to reduce toxicity enhances ADC efficacy
- CD276 ADCs are highly effective against HER2-negative breast cancers

## INCLUSION AND DIVERSITY

We support inclusive, diverse, and equitable conduct of research.
