# Supplementary material for: Engineering CD276/B7-H3-targeted antibody-drug conjugates with enhanced cancer-eradicating capability
Source: Cell Rep. Author manuscript; Available in PMC 2024 Feb 16. (PMC10872261; doi:10.1016/j.celrep.2023.113503)
Supplement: 1 [file NIHMS1954777-supplement-1.pdf]

Cell Reports, Volume *42*

## Supplemental information

# Engineering CD276/B7-H3-targeted antibody-drug conjugates with enhanced cancer-eradicating capability

**Yang Feng, Jaewon Lee, Liping Yang, Mary Beth Hilton, Karen Morris, Steven Seaman, Veera V. Shivaji R. Edupuganti, Kuo-Sheng Hsu, Christopher Dower, Guojun Yu, Daeho So, Pradip Bajgain, Zhongyu Zhu, Dimiter S. Dimitrov, Nimit L. Patel, Christina M. Robinson, Simone Difilippantonio, Marzena Dyba, Amanda Corbel, Falguni Basuli, Rolf E. Swenson, Joseph D. Kalen, Sreedhar Reddy Suthe, Myer Hussain, James S. Italia, Colby A. Souders, Ling Gao, Martin J. Schnermann, and Brad St. Croix**

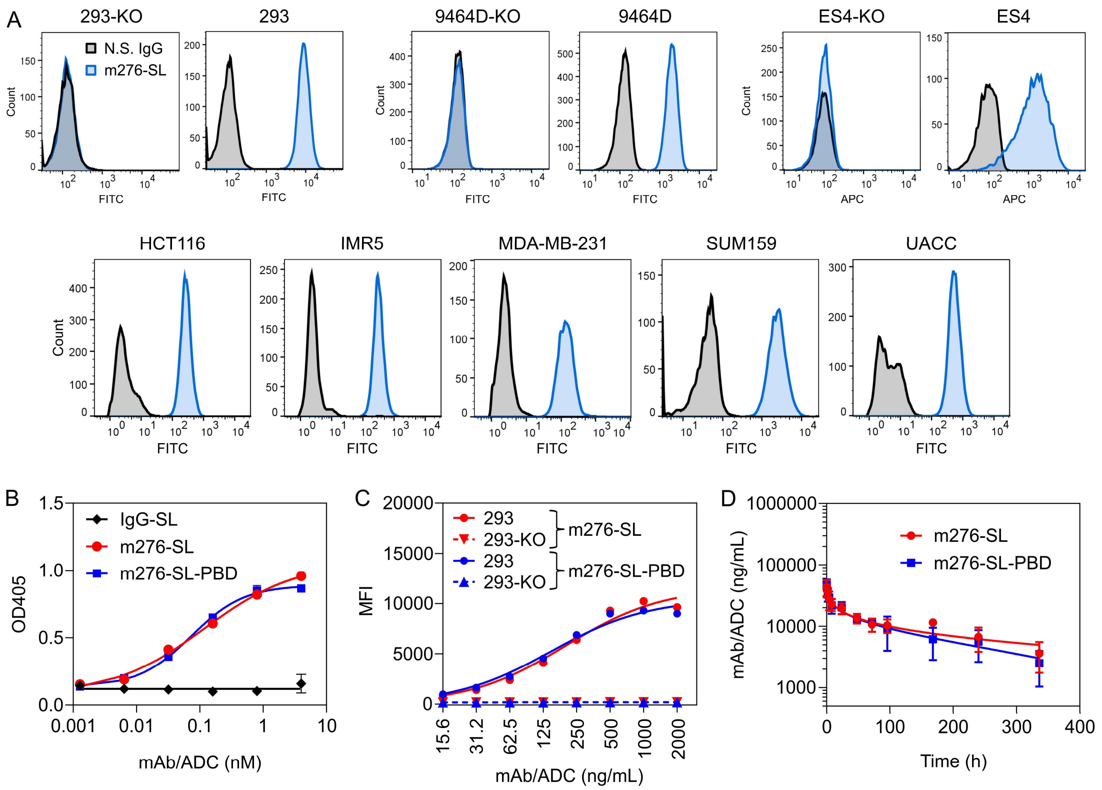


E

| | Cmax | $AUC_{0\text{-last}}$ (µg•h/mL) | $AUC_{0\text{-}\infty}$ (µg•h/mL) | tmax (h) | λz (/h) | $t_{1/2}$ (h) |
|---|---|---|---|---|---|---|
| m276-SL | 43.6 | 3423.0 | 3994.1 | 0.1 | 0.006 | 109.1 |
| m276-SL-PBD | 51.3 | 2917.7 | 3210.3 | 0.1 | 0.009 | 81.4 |

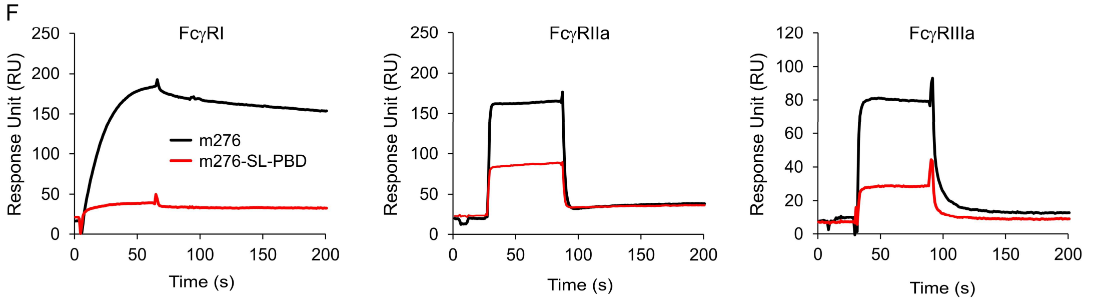


***Supplementary Figure S1, Related to Figure 1. Characterizing m276-SL-PBD and its parental mAbs: in vitro binding to CD276 and FcγR, and in vivo pharmacokinetics.***

**(A)** Flow cytometry was used to measure binding of nonspecific IgG or m276-SL the cell lines indicated. CRISPR/Cas9 gene editing was used to knockout CD276 in the 293-KO, 9464D-KO and ES4-KO cells. **(B)** An ELISA was used to monitor binding of m276-SL monoclonal antibody (mAb) and m276-SL-PBD antibody-drug conjugate (ADC) to immobilized recombinant CD276 ectodomain. Error bars denote SD. **(C)** Flow cytometry was use to assess dose-dependent binding of m276-SL and m276-SL-PBD to 293 (CD276 wildtype) and 293-KO (CD276 knockout) cells. **(D)** An ELISA was used to monitor antibody concentrations in serum following a single i.v. injection of 2.5 mg/kg m276-SL or m276-SL-PBD. Error bars denote SD. **(E)** Table summarizing the pharmacokinetic properties of m276-SL or m276-SL-PBD from the experiment shown in (D). (**F**) SPR analysis of m276 parent antibody and the m276-SL-PBD binding to Fc-gamma receptors. m276-SL-PBD contains the Fc blocking LALAPG mutations.

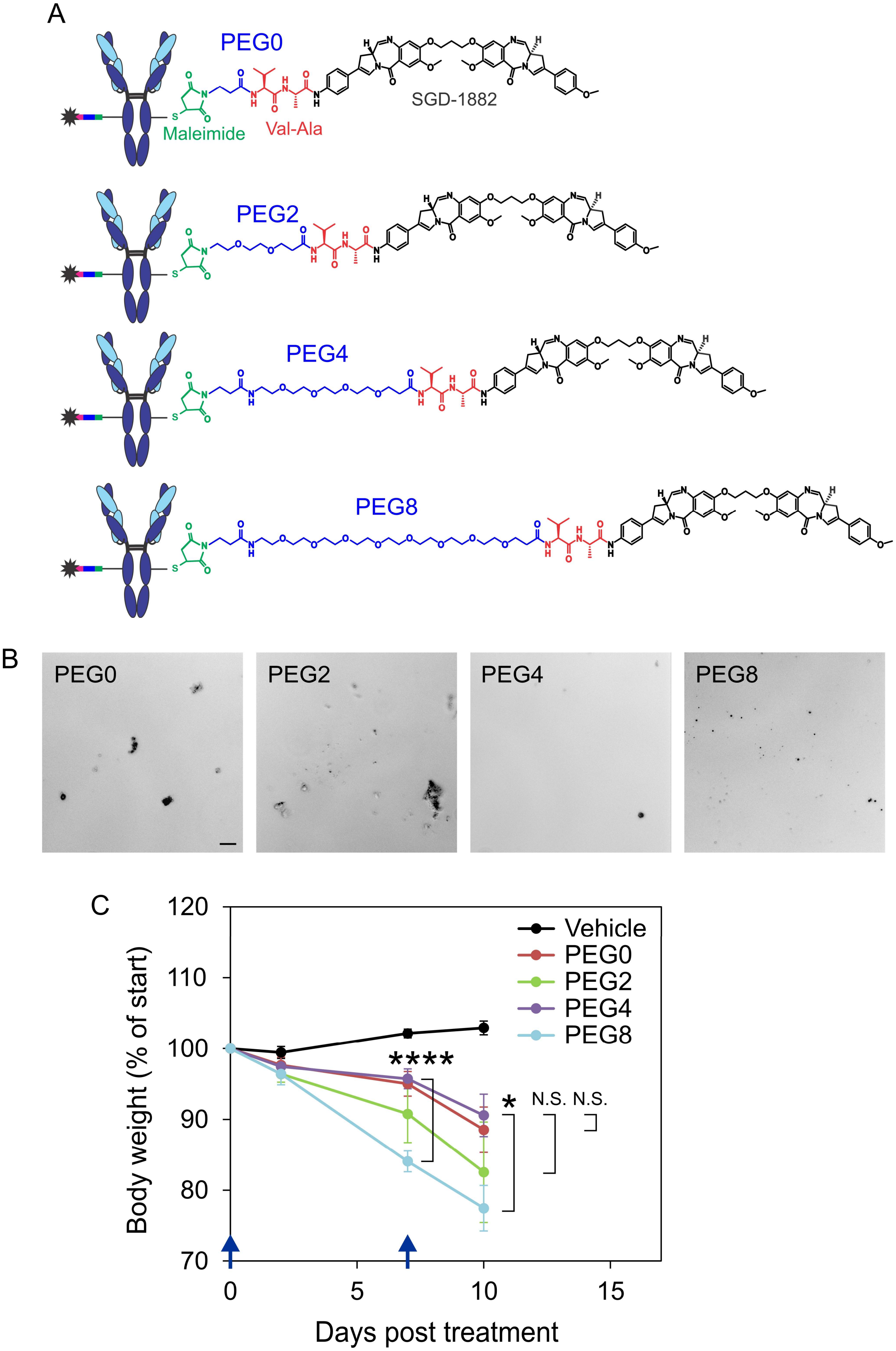
A
PEG0
Maleimide
Val-Ala
SGD-1882
PEG2
PEG4
PEG8
B
PEG0
PEG2
PEG4
PEG8
C
Vehicle
PEG0
PEG2
PEG4
PEG8
Body weight (% of start)
Days post treatment
****
*
N.S.
N.S.
120
110
100
90
80
70
0
5
10
15

***Supplementary Figure S2, Related to Figure 1. PEG4 drug-linkers, with improved solubility and performance, do not increase toxicity in vivo.***

**(A)** Chemical structure of the PEG0, PEG2, PEG4 and PEG8 drug linkers tested. **(B)** Brightfield images of the PEG0, PEG2, PEG4 and PEG8 drug linkers shown in A after dissolving in propylene glycol. Bar = 20 μm. **(C)** Body weight analysis following two injections (blue arrows) of vehicle (control) or a high dose (4 mg/kg) of m276-SL-PBD containing PEG0, PEG2, PEG4 or PEG8. ****; $p \leq 0.0001$, *$p \leq 0.05$. N.S.: non-significant. Error bars denote SD.

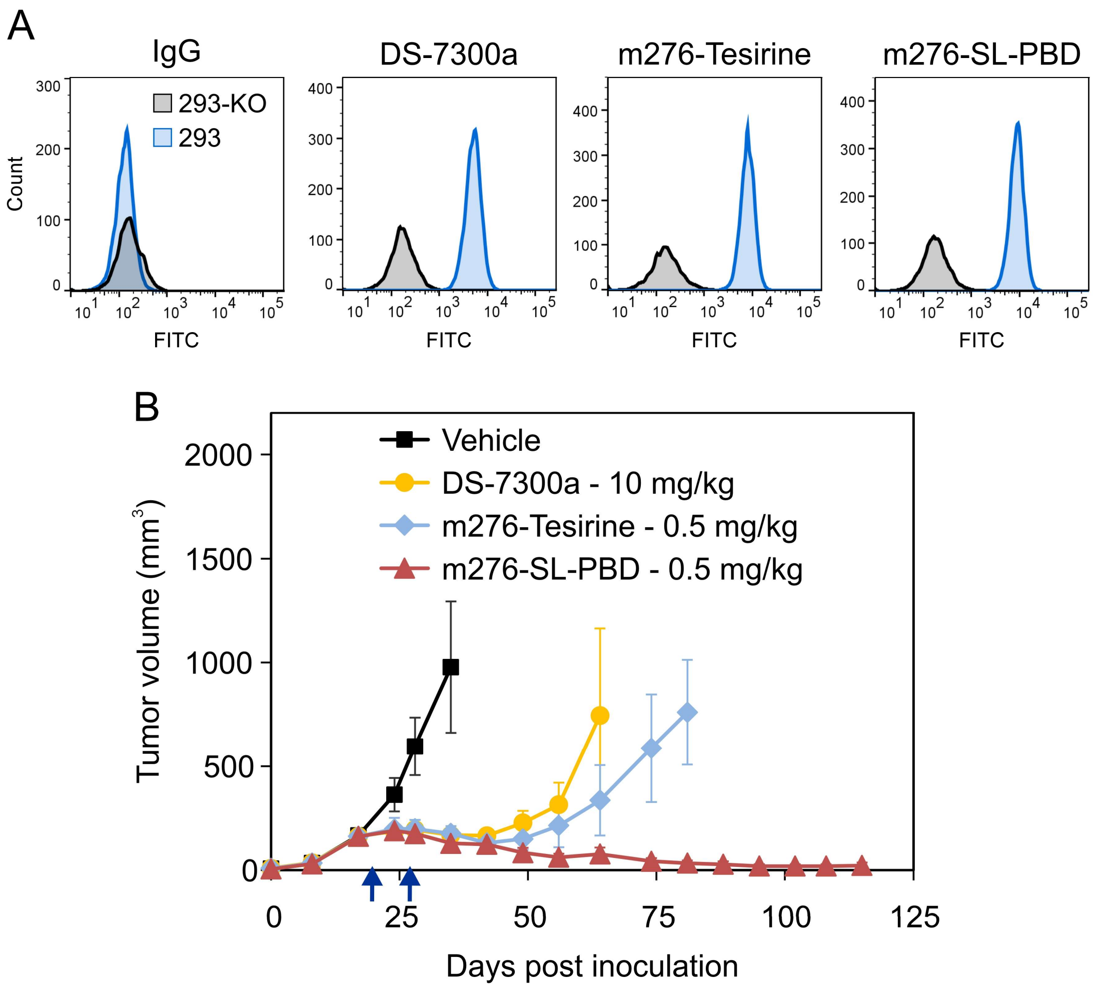


***Supplementary Figure S3, Related to Figure 1. Comparison of CD276 ADCs with different payloads.***

**(A)** Flow cytometry was used to measure binding of nonspecific IgG, or the CD276 ADCs DS-7300a, m276-Tesirine or m276-SL-PBD (containing talirine) to 293 wildtype or CD276 KO cells. **(B)** Subcutaneous growth of HCT-116 colon tumors in response to two treatments (blue arrows) with vehicle (control), 10 mg/kg of DS-7300a, 0.5 mg/kg of m276-Tesirine, or 0.5 mg/kg of m276-SL-PBD (containing talirine). n = 8-14/group. Error bars denote SEM.

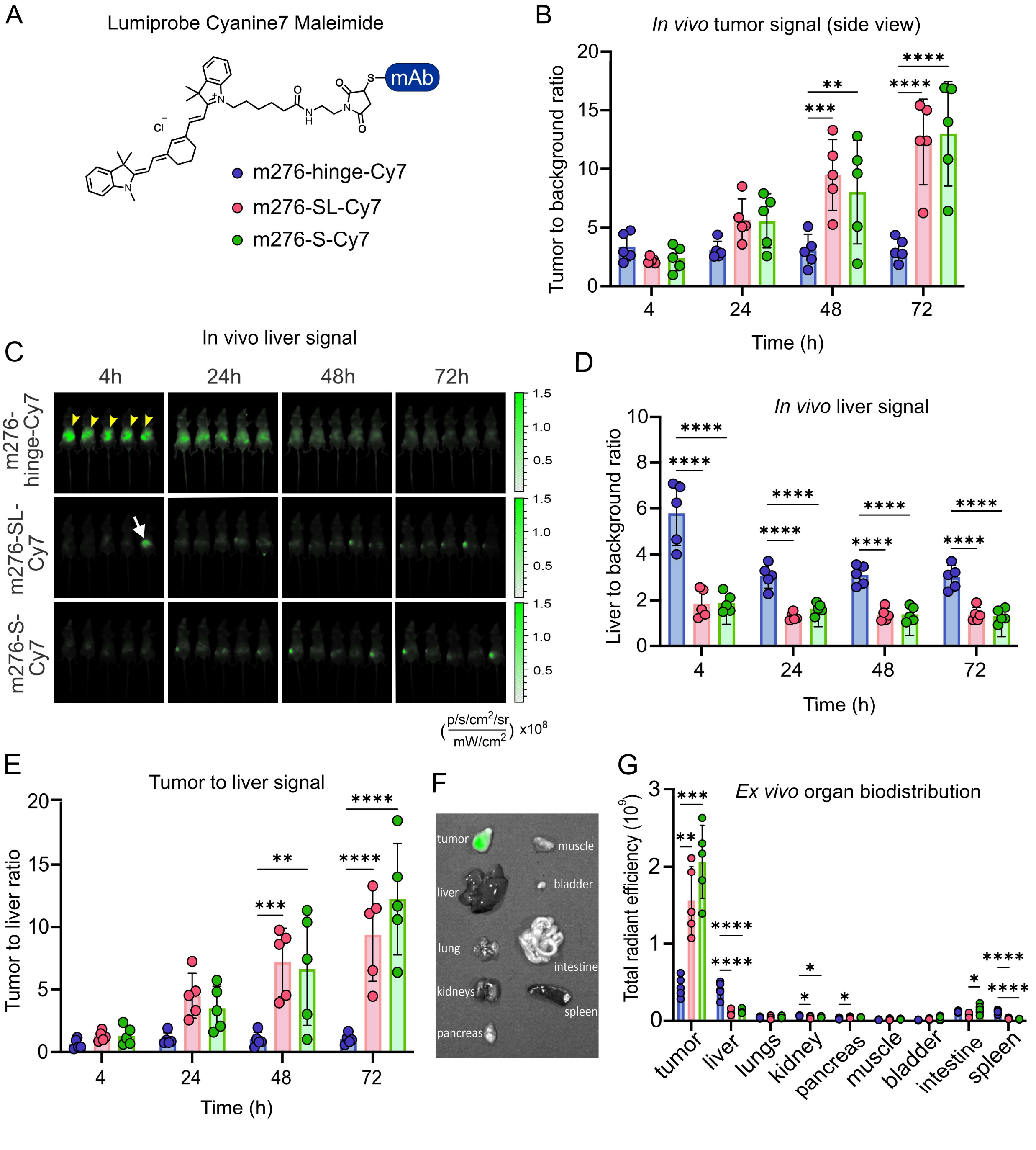

A
Lumiprobe Cyanine7 Maleimide
mAb
m276-hinge-Cy7
m276-SL-Cy7
m276-S-Cy7
B
In vivo tumor signal (side view)
Tumor to background ratio
Time (h)
C
In vivo liver signal
4h
24h
48h
72h
m276-hinge-Cy7
m276-SL-Cy7
m276-S-Cy7
D
In vivo liver signal
Liver to background ratio
Time (h)
E
Tumor to liver signal
Tumor to liver ratio
Time (h)
F
tumor
liver
lung
kidneys
pancreas
muscle
bladder
intestine
spleen
G
Ex vivo organ biodistribution
Total radiant efficiency ($10^9$)
tumor
liver
lungs
kidney
pancreas
muscle
bladder
intestine
spleen

***Supplementary Figure S4, Related to Figure 2. Fluorescence imaging to monitor impact of labeling method on m276 distribution.***

**(A)** Chemical structure of imagable Lumiprobe Cy7 maleimide dye used to label the endogenous hinge cysteines of m276 (m276-hinge-Cy7) or the free cysteine at S239C on m276-SL (m276-SL-Cy7) and m276-S (m276-S-Cy7). **(B)** Quantification of the tumor to background ratio of each of the m276-hinge-Cy7, m276-SL-Cy7, and m276-S-Cy7 antibodies shown in figure 2A (main text). **(C)** *In vivo* fluorescent imaging of the liver signal (ventral view) from Cy7 labeled m276 antibodies in JIMT tumor-bearing mice at 4, 24, 48 and 72 h post injection. Following m276-hinge-Cy7 injection a prominent signal is detected in the liver by 4 h (yellow arrowheads). Some bleed-through signal from the tumor on the dorsal flank can be observed in mice treated with m276-SL-Cy7 and m276-S-Cy7 (for example, see white arrow). n=5 **(D)** Quantification of the liver to background ratio of each of the Cy7 labeled m276 antibodies shown in (C). **(E)** Quantification of the tumor to liver ratio of m276-hinge-Cy7, m276-SL-Cy7, and m276-S-Cy7. **(F)** *Ex vivo* fluorescent images of organs and tissues taken 72 h postinjection of the Cy7 labeled m276 antibodies. A representative image from a mouse injected with m276-SL-Cy7 is shown. **(G)** *Ex vivo* quantification of the Cy7 fluorescence signal in each of the organs or tissues was assessed at 72 h post-injection. Data points are displayed as mean ± SD, and the p-values were evaluated by ANOVA. * p-value ≤ 0.05, ** p-value ≤ 0.01, *** p-value ≤ 0.001, **** p-value ≤ 0.0001.

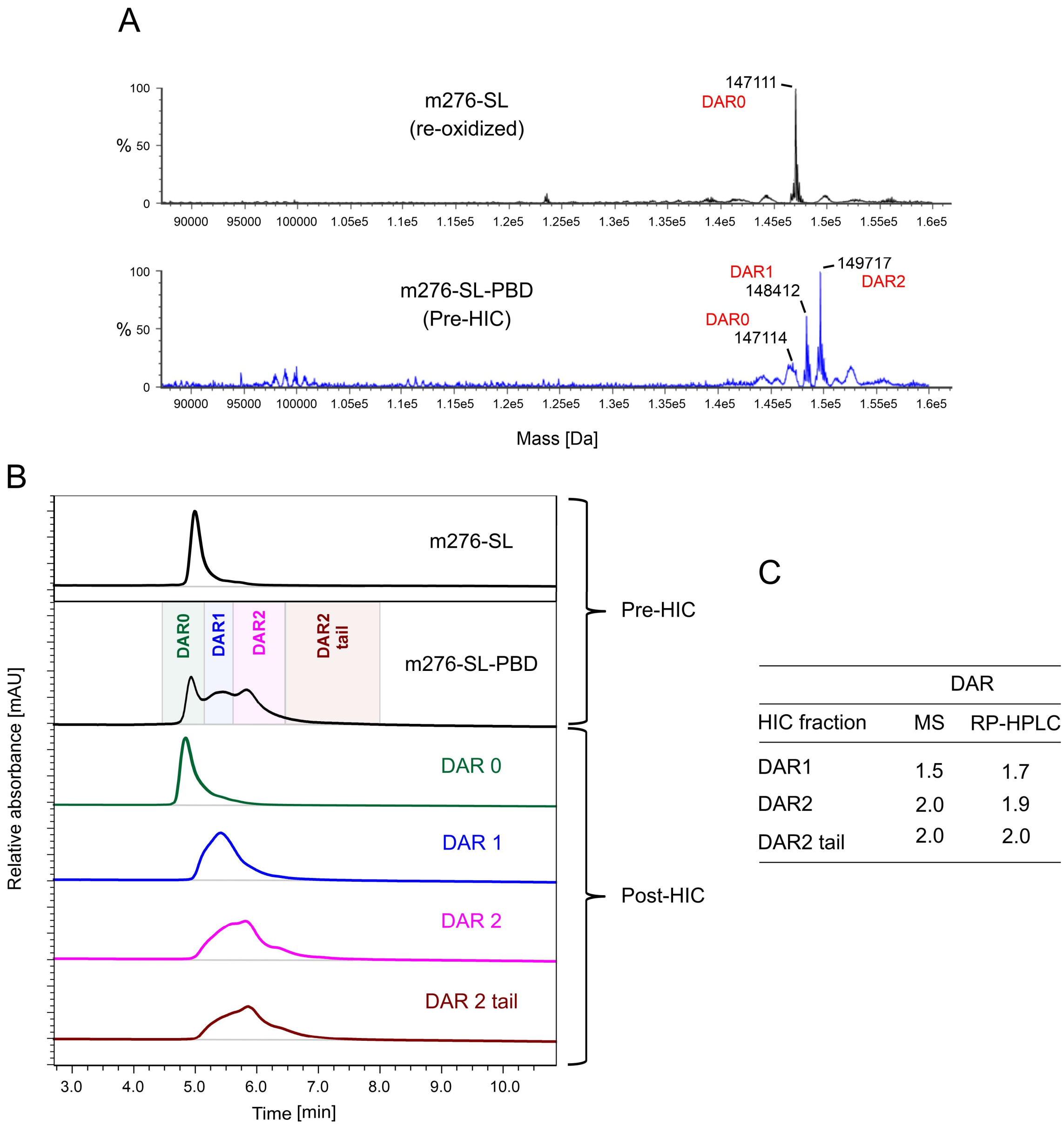


C

| | DAR | |
|---|---|---|
| HIC fraction | MS | RP-HPLC |
| DAR1 | 1.5 | 1.7 |
| DAR2 | 2.0 | 1.9 |
| DAR2 tail | 2.0 | 2.0 |

***Supplementary Figure S5, Related to Figure 2. HIC purification enhances PBD payload labeling.***

**(A)** LC-MS was used to monitor the DAR on m276-SL after re-oxidation (top panel) or following PBD conjugation (Pre-HIC). **(B)** Preparative HIC was used to fractionate the labeled m276-SL-PBD and isolate samples enriched for increasing hydrophobicity: DAR 0, DAR 1, DAR 2 and DAR 2 tail. Following HIC purification (Post-HIC), analytical HIC was performed to verify the purity of the isolated fractions. For comparison, the HIC profile of the parent m276-SL antibody prior to drug labeling is shown at the top. Note that the DAR2 tail fraction displayed a chromatogram similar to the DAR2 fractions upon post-HIC analysis indicating that the “tailing” was transient and reversible and unlikely due to PBD overlabeling. **(C)** DAR calculations based on mass spectromety (MS) and reverse-phase high-performance liquid chromatography (RP-HPLC).

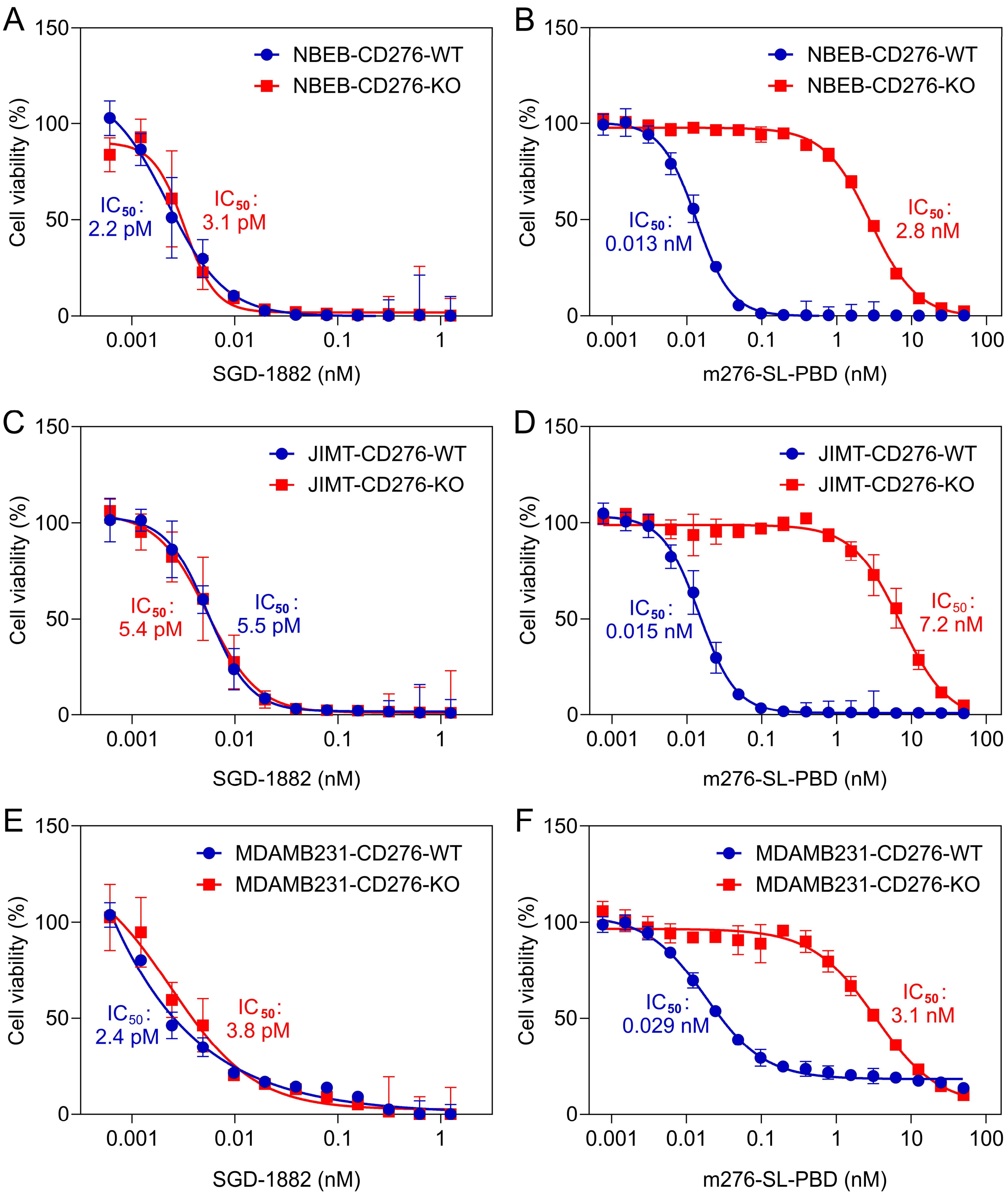


***Supplementary Figure S6, Related to Figure 4. Cytotoxic response to treatment with m276-SL-PBD, but not PBD free drug, depends on CD276 target expression.***

**(A-F)** Cell viability assays were used to measure the cytotoxicity of SGD-1882 free drug (A, C, E) or m276-SL-PBD ADC (B, D, F) against CD276 WT and KO cells derived from NB-EB neuroblastoma (A, B), JIMT breast cancer (C, D) and MDA-MB-231 breast cancer cells (E,F). Note that target expressing CD276 WT cells are >100-fold more sensitive to the m276-SL-PBD ADC than CD276 KO cells. Error bars denote SD.

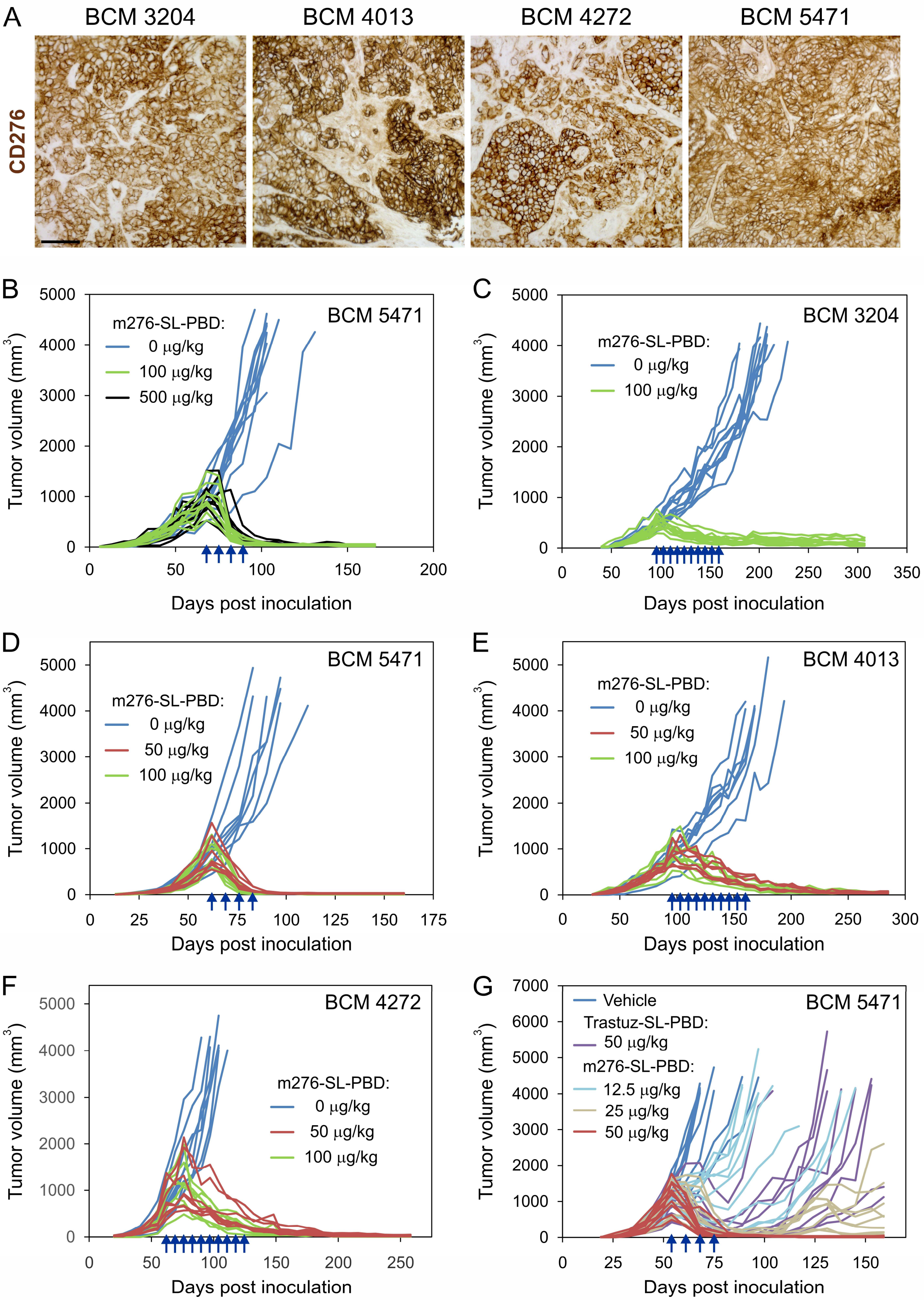
A
BCM 3204
BCM 4013
BCM 4272
BCM 5471
CD276
B
BCM 5471
m276-SL-PBD:
0 μg/kg
100 μg/kg
500 μg/kg
Tumor volume (mm3)
Days post inoculation
C
BCM 3204
m276-SL-PBD:
0 μg/kg
100 μg/kg
Tumor volume (mm3)
Days post inoculation
D
BCM 5471
m276-SL-PBD:
0 μg/kg
50 μg/kg
100 μg/kg
Tumor volume (mm3)
Days post inoculation
E
BCM 4013
m276-SL-PBD:
0 μg/kg
50 μg/kg
100 μg/kg
Tumor volume (mm3)
Days post inoculation
F
BCM 4272
m276-SL-PBD:
0 μg/kg
50 μg/kg
100 μg/kg
Tumor volume (mm3)
Days post inoculation
G
BCM 5471
Vehicle
Trastuz-SL-PBD:
50 μg/kg
m276-SL-PBD:
12.5 μg/kg
25 μg/kg
50 μg/kg
Tumor volume (mm3)
Days post inoculation

***Supplementary Figure S7, Related to Figure 6. Low dose m276-SL-PBD evokes durable tumor regression in multiple CD276$^+$ breast cancer PDX models.***

**(A)** Immunohistochemistry was used to evaluate CD276 expression in breast cancer patient derived xenografts. Scale bar: 100μm. **(B-G)** Orthotopic growth of BCM5471 (B,D,G), BCM3204 (C), BCM4013 (E) and BCM4272 (F) breast tumors in response to treatment with vehicle alone (0 μg/kg), or the indicated dose of m276-SL-PBD. Treatments with m276-SL-PBD were initiated when average tumor volumes reached ~500 mm$^3$ (C) or ~1000 mm$^3$ (B, D-G) and were administered once per week on the days shown (blue arrows). n = 7-8/group. Individual growth curves are shown. The data from D, E and F represent the individual growth curves that correspond to average growth curves of Figure 6 C, D and E of the main text while the data from G represent individual growth curves for figure 7.
